# Manipulating Endoplasmic Reticulum-Plasma Membrane Tethering in Plants Through Fluorescent Protein Complementation

**DOI:** 10.3389/fpls.2019.00635

**Published:** 2019-05-22

**Authors:** Kai Tao, Justin R. Waletich, Felipe Arredondo, Brett M. Tyler

**Affiliations:** ^1^Molecular and Cellular Biology Program, Oregon State University, Corvallis, OR, United States; ^2^Department of Botany and Plant Pathology, Oregon State University, Corvallis, OR, United States; ^3^Center for Genome Research and Biocomputing, Oregon State University, Corvallis, OR, United States

**Keywords:** tethering, plasma membrane, endoplasmic recticulum, peripheral membrane protein, integral membrane protein

## Abstract

The bimolecular fluorescence complementation (BiFC) assay has been widely used to examine interactions between integral and peripheral proteins within putative plasma membrane (PM) microdomains. In the course of using BiFC assays to examine the co-localization of plasma membrane (PM) targeted receptor-like kinases (RLKs), such as FLS2, with PM micro-domain proteins such as remorins, we unexpectedly observed heterogeneous distribution patterns of fluorescence on the PM of *Nicotiana benthamiana* leaf cortical cells. These patterns appeared to co-localize with the endoplasmic reticulum (ER) and with ER-PM contact sites, and closely resembled patterns caused by over-expression of the ER-PM tether protein Synaptotagmin1 (SYT1). Using domain swap experiments with SYT1, we inferred that non-specific dimerization between FLS2-VenusN and VenusC-StRem1.3 could create artificial ER-PM tether proteins analogous to SYT1. The same patterns of ER-PM tethering were produced when a representative set of integral membrane proteins were partnered in BiFC complexes with PM-targeted peripheral membrane proteins, including PtdIns(4)P-binding proteins. We inferred that spontaneous formation of mature fluorescent proteins caused the BiFC complexes to trap the integral membrane proteins in the ER during delivery to the PM, producing a PM-ER tether. This phenomenon could be a useful tool to deliberately manipulate ER-PM tethering or to test protein membrane localization. However, this study also highlights the risk of using the BiFC assay to study membrane protein interactions in plants, due to the possibility of alterations in cellular structures and membrane organization, or misinterpretation of protein-protein interactions. A number of published studies using this approach may therefore need to be revisited.

## Significance Statement

The bimolecular fluorescence complementation (BiFC) assay has been widely used to examine interactions between integral and peripheral proteins within putative plasma membrane (PM) microdomains. Using domain swap experiments involving the endoplasmic reticulum-PM tether protein SYT1, we have obtained evidence that BiFC complexes involving one integral membrane protein and one peripheral membrane protein can act as synthetic EM-PM tethers, producing images that resemble microdomain co-localization, but are actually artifacts; a number of published studies should thus be revisited.

## Introduction

A variety of internal organelles and structures are closely associated with the PM, especially in plant cells where the large central vacuole compresses the cytoplasm into a thin layer against the PM. These include endosomes, multi-vesicular bodies (MVBs), cortical microtubules, actin filaments, and cortical layers of the endoplasmic reticulum (ER). The ER is the largest membrane-bound organelle comprising an expansive network throughout the cell, functioning in protein synthesis and modification, lipid biosynthesis, metabolism, and Ca^2+^ and other intracellular signaling (Burgoyne et al., 2015). In order to coordinate its specialized functions with other membrane-bound organelles or the PM, the ER is functionally connected through vesicular trafficking, which involves the fusion of the membranes of interacting organelles. As an alternative mechanism of communication, tethering structures known as membrane contact sites (MCSs) provide a transient or durable “bridge” between the ER and other organelles including the PM. MCSs maintain the participating membranes in close proximity without membrane fusion taking place, enabling inter-organelle communication and exchange of metabolites (Helle et al., 2013; Perez-Sancho et al., 2016). MCSs formed by the PM and the ER are called ER-PM contact sites (EPCSs). These sites have been characterized in a variety of eukaryotic species, including animals, yeast, and plants (Saheki and De Camilli, 2017; Wang et al., 2017). EPCSs are precisely maintained and regulated by a variety of protein tethers or protein complexes. Though it has been suggested that each of these tethers or protein groups is associated with different cellular functions, all of them share the same structural characteristics: an ER-anchored region that can be either a hydrophobic hairpin inserted into the ER membrane or a transmembrane domain (TMD) integrated into the ER, combined with a cytosolic domain that contains motifs for binding lipids or proteins in the PM (Prinz, 2014; Saheki and De Camilli, 2017; Wang et al., 2017).

In plants, two different major protein groups sharing these common features have been identified as tethers at EPCSs, namely Synaptogamins (SYTs) and Vesicle-associated membrane protein-Associated Proteins (VAPs). One well-characterized tether protein is *Arabidopsis* Synaptogamin 1 (SYT1, also known as SYTA). SYT1 contains a TMD at the N terminus, a synaptotagmin-like-mitochondria-lipid binding domain (SMP) close to the TMD, and a C-terminal cytoplasmic domain containing two conserved calcium-binding domains C2A and C2B that are responsible for binding a variety of negatively charged phospholipids on the PM (Schapire et al., 2008; Yamazaki et al., 2010; Levy et al., 2015; Pérez-Sancho et al., 2015). The TMD mediates ER-anchoring and the C2AC2B domain mediates PM-binding, together conferring the ER-PM tethering function of SYT1. Plant EPCSs have also been shown to contain VAP27-1 and VAP27-3 which are homologous to mammalian VAPs (Wang et al., 2014, 2016). The C-terminal domains of the VAP27s are examples of integral membrane tail-anchoring domains, and anchor the VAP27s to the ER membrane. PM-binding of the VAP27s is via interaction of their N-terminal conserved major sperm domain (MSD) with the NETWORKED protein, NET3C, as well as cytoskeletal elements. NET3C is plant-specific and located in EPCSs. In addition, VAP27s were also found to form protein complexes with plant oxysterol-binding-protein-related proteins (ORPs), which contain pleckstrin homology (PH) domains for the binding of phosphoinositides (PIPs) in the PM (Saravanan et al., 2009). Furthermore, VAP27 was recently shown to directly interact with clathrin and with PIPs at endocytic subdomains in the PM, resulting in the establishment of tethering (Stefano et al., 2018).

The bimolecular fluorescence complementation (BiFC) assay (Kerppola, 2008) is a commonly used experimental approach to study protein-protein interactions (Kerppola, 2008). The BiFC assay is based on two non-fluorescing fragments split from a fluorescent protein, each of which is translationally fused with a different protein of interest; interaction between the proteins of interest will bring the two non-fluorescing fragments into proximity with each other resulting in the re-assembly of a functional fluorescent protein (Kerppola, 2006). Thus, the BiFC assay not only enables identification of a potential protein-protein interaction, but also allows direct visualization of the protein complex *in vivo*. Due to these useful characteristics, the BiFC assay has also been successfully applied as a high-throughput approach in several large-scale studies to map potential protein-protein interactions (Remy and Michnick, 2004; Boruc et al., 2010; Snider et al., 2013). Venus, a variant of enhanced yellow fluorescence protein (EYFP) with a higher efficiency of maturation and better adaptability in acid and high temperature environments, has become a widely used fluorescent protein for BiFC assays (Saka et al., 2007; Kodama and Hu, 2010; Miller et al., 2015). Moreover, a residue at position 155 has proven useful as a split site for Venus in BiFC assays (Wong and O'Bryan, 2011; Kodama and Hu, 2012). However, a challenge for this strategy is the spontaneous reassembly of the two fragments in the absence of associating protein partners that can result in false positive BiFC signals (Shyu et al., 2006; Zamyatnin et al., 2006; Saka et al., 2007).

Growing evidence has revealed that different phospholipid species and membrane proteins in the PM may be organized into coalescences with diameters ranging from 2.0 to 300 nm, referred to as “lipid or membrane rafts” or more recently as “microdomains” (Kusumi et al., 2011; Lillemeier and Klammt, 2012; Varshney et al., 2016). These microdomains are the result of lipid-lipid, protein-lipid, and protein-protein interactions in the plasma membrane, potentially providing functional platforms to orchestrate a multitude of signaling pathways (Kusumi et al., 2012). To date, two major protein families, called flotillins and remorins have been associated with plasma membrane microdomains (Raffaele et al., 2009). Flotillins are widely present in all kingdoms of life, and their membrane targeting is mediated by either myristoylation, palmitoylation, or both (Jarsch et al., 2014). In contrast, remorins are plant-specific proteins, which have been well-characterized and contain a highly conserved C-terminal coiled-coil domain for plasma membrane anchoring (Perraki et al., 2012).

In plants, a spectrum of PM-bound receptor-like kinases (RLKs) are employed to coordinate signaling pathways in growth, development, and innate immunity (He et al., 2018). Several RLKs have been found to be functionally associated with remorins or flotillins (Jarsch et al., 2014). For example, the remorin protein MtSYMREM1 from the legume *Medicago truncatula*, was reported to function as a scaffolding protein mediating spatial distribution of several RLKs during symbiotic plant-microbe interactions (Lefebvre et al., 2010), including MtNFP (Arrighi et al., 2006), MtLYK3 (Smit et al., 2007), and MtDMI2 (Limpens et al., 2005). Likewise, the closest *Lotus japonicus* homolog of MtSYMREM1, LjSYMREM1 (Tóth et al., 2012), was shown to interact with the *L. japonicus* RLKs, LjNFR5 (Madsen et al., 2003), LjNFR1 (Radutoiu et al., 2003), and LjSYMRK (Stracke et al., 2002). The *Arabidopsis* flotillin protein, AtFlotillin1 (Borner et al., 2005), was shown to be critically involved in the activation of the RLK growth regulator, AtBRI1 (Russinova et al., 2004); the two proteins showed increased co-localization in response to the brassinosteroid ligand (Wang et al., 2015). More recently, Bücherl et al. (2017) observed that in *Arabidopsis*, AtBRI1 and the RLK immune receptor AtFLS2 (Gómez-Gómez and Boller, 2000) are heterogeneously but differently distributed in the membrane and that each receptor was associated with distinct remorin proteins. Despite these advances, the underlying mechanisms of compartmentalization of cell surface receptors into plasma membrane microdomains is still incompletely understood.

In this study, we set out to investigate pairwise associations between a set of representative membrane receptors, remorins and lipid-binding proteins using the BiFC assay. When RLKs such as FLS2 were ectopically co-expressed with *Solanum tuberosum* remorin StRem1.3 in *N. benthamiana* cortical cells, the BiFC fluorescent signal was heterogeneously distributed in distinct patch-like domains or nearly continuous networks across the PM. Co-localization assays suggested that these patterns may be associated with ER-PM contact sites, and thus that the BiFC complexes might unexpectedly be acting as artificial ER-PM tethering proteins. Here, using domain swap experiments involving the tether protein SYT1, we have obtained evidence that any BiFC complex that involves one integral membrane protein and one peripheral membrane protein has the potential to act as an artificial ER-PM tethering protein. This artifact has been overlooked in previous studies of membrane organization using BiFC assays.

## Results

### Heterogeneous Patch-Like Distribution of FLS2-StRem1.3 BiFC Complexes

**Figure 1:**
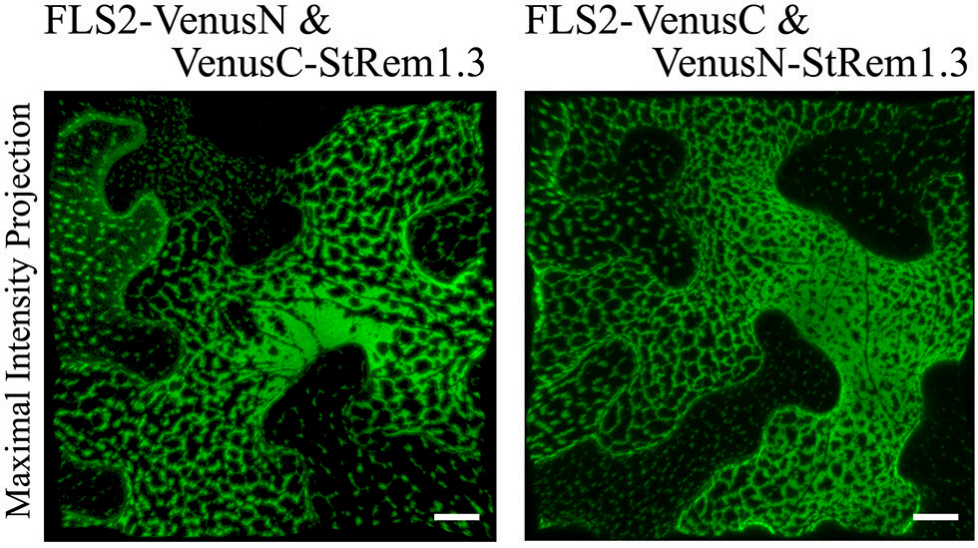
Heterogeneous distribution of FLS2-StRem1.3 BiFC complexes in *N. benthamiana* leaf cortical cells. Examples of BiFC fluorescence signals observed after co-expression of the following pairs of constructs: FLS2-VenusN and VenusC-StRem1.3; FLS2-VenusC and VenusN-StRem1.3. Scale bars represent 10 μm.

To provide a positive control for our studies of protein-lipid organization in the PM using BiFC, we first chose FLS2 and StRem1.3, which have been reported to co-localize with each other at the haustorial interface when FLS2 is activated by flg22 (Bozkurt et al., 2015). To do this, we fused the N-terminal fragment of Venus (VenusN) to the C terminus of FLS2, and the C-terminal fragment of Venus (VenusC) to the N terminus of StRem1.3. We also made complementary FLS2-VenusC and VenusN-StRem1.3 constructs. The pairs of BiFC constructs were ectopically co-expressed under the control the Cauliflower Mosaic Virus (CaMV) 35S promoter in *N. benthamiana* leaf cortical cells using *Agrobacterium tumefaciens*-mediated transient transformations. By confocal microscopy live-cell imaging, we observed strong BiFC fluorescence signals with both BiFC configuration pairs: FLS2-VenusN + VenusC-StRem1.3 and FLS2-VenusC + VenusN-StRem1.3 (Figure 1). In both configurations, the BiFC fluorescence signal was heterogeneously distributed in distinct patterns ranging from discrete patches through to continuous networks spanning the cortical surface. As a control, we co-expressed the FLS2 and StRem1.3 fusions with complementary Venus fragments that were not fused to another protein. In each case, we observed appreciable BiFC fluorescent signals (Supplementary Figure S1), indicating that non-specific interactions between the two fragments of Venus could occur in the absence of FLS2-StRem1.3 associations (Kodama and Hu, 2010; Gookin and Assmann, 2014). However, the BiFC fluorescence signals produced by each of these control pairs were homogenously distributed on the plasma membrane, especially in the case of FLS2. In each case, the subcellular localization closely matched that of fusions of FLS2 or StRem1.3 with full-length YFP (Supplementary Figure S1), indicating that the subcellular localization of each control BiFC complex was determined by the respective FLS2 or StRem1.3 component. It also indicated that the heterogeneous distribution patterns observed with the FLS2-StRem1.3 BiFC complexes were produced only when both FLS2 and StRem1.3 were present. Similar results were obtained when using YFP as the BiFC fluorophore (Supplementary Figure S2), or when the constructs were expressed in *Arabidopsis* mesophyll protoplasts (Supplementary Figure S2). When StRem1.3 was replaced by a mutant, StRem1.3^*^ that could not bind the PM, the BiFC complexes displayed the localization expected for FLS2 alone (Supplementary Figures S1B,C). StRem1.3^*^ contains mutations in the membrane-insertion loop of StRem1.3 that abolish the hydrophobicity of the loop (Perraki et al., 2012).

### FLS2-StRem1.3 BiFC Complexes Appear to Localize to ER-PM Contact Sites

**Figure 2:**
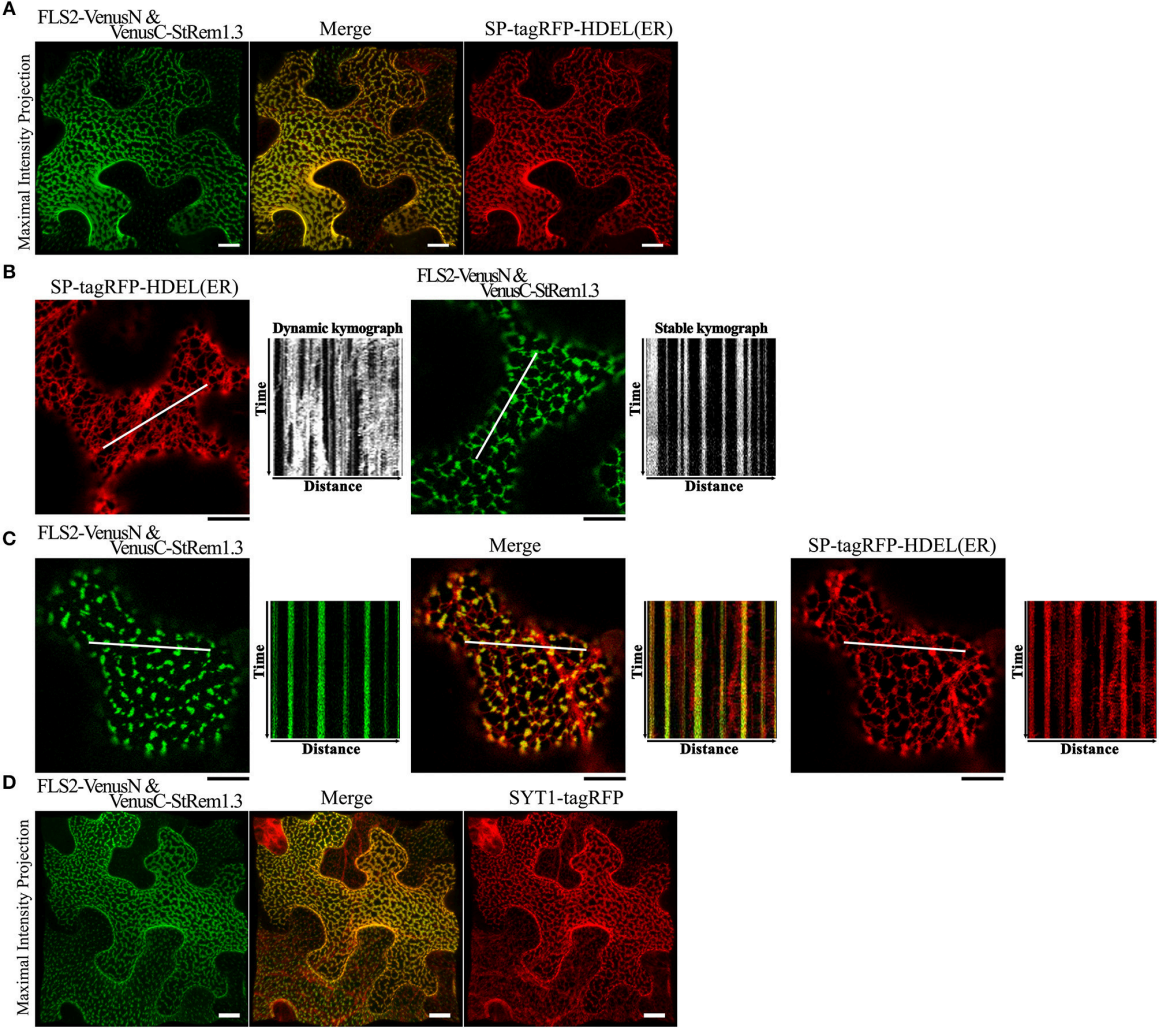
FLS2-StRem1.3 BiFC complexes appear to associate with ER-PM contact sites in *N. benthamiana* leaf cortical cells. **(A)** FLS2-StRem1.3 BiFC complexes co-expressed with endoplasmic reticulum maker SP-tagRFP-HDEL. **(B)** Dynamic motion of the ER network labeled by ER SP-tagRFP-HDEL and stable localization of puncta of FLS2-StRem1.3 BiFC complexes revealed by kymograph analysis. **(C)** Kymograph analysis of FLS2-StRem1.3 BiFC complexes co-expressed with SP-tagRFP-HDEL. In **(B,C)**, kymographs were created from a short time-lapse series (80 s) across transect lines ~30 μm in length (shown in white). **(D)** FLS2-StRem1.3 BiFC complexes co-expressed with *Arabidopsis* ER-PM tethering protein Synaptogamin 1 (SYT1) fused to tagRFP. All scale bars (white in **A,D**; black in **B,C**) represent 10 μm.

Since the net-like distribution of the FLS2-StRem1.3 BiFC complexes in many cells resembled the distribution of the cortical ER, we performed co-localization assays using the ER marker SP-tagRFP-HDEL (Matsushima et al., 2002). The cellular distribution of FLS2-StRem1.3 BiFC fluorescence closely followed the distribution patterns of SP-tagRFP-HDEL, namely net-like and sheet-like patterns of fluorescence (Figure 2A). We also commonly observed dynamic movements of the ER network (Stefano et al., 2014) when SP-tagRFP-HDEL was expressed alone (Figure 2B, Supplementary Movie S1). We documented the dynamic movements of the labeled organelles by using kymographs, in which the fluorescent signal along a transect is imaged over time. As shown in Figure 2B, the dynamic movements of the ER network labeled by SP-tagRFP-HDEL produced a chaotic kymograph. In contrast, we observed that the puncta of the FLS-StRem1.3 BiFC complexes were relatively static, producing straight lines on the kymograph (Figure 2B, Supplementary Movie S2). Moreover, in cells co-expressing both FLS2-StRem1.3 BiFC complexes and SP-tagRFP-HDEL, the puncta of the FLS2-StRem1.3 BiFC complexes co-localized with SP-tagRFP-HDEL at junctions in the ER network where the SP-tagRFP-HDEL signal showed increased stability (Figure 2C). However, small portions of the ER networks that were not co-localized with FLS2-StRem1.3 complexes still showed dynamic movements (Figure 2C). Since ER-PM contact sites are sites of reduced mobility of the ER (Henne et al., 2015), we hypothesized that the FLS2-StRem1.3 puncta may correspond to ER-PM contact sites.

To test whether the FLS2-StRem1.3 puncta may correspond to ER-PM contact sites, we co-expressed tagRFP-tagged SYT1 protein from *Arabidopsis*, which has been well-characterized as a tethering protein for ER-PM contact sites (Pérez-Sancho et al., 2015). As shown in Figure 2D, FLS2-StRem1.3 BiFC signals were clearly co-localized with SYT1. Moreover, a characteristic property of ER-PM junctions in mammalian cells is that they restrict the distribution of other membrane proteins in mammalian cells (Carrasco and Meyer, 2011). Though the exclusion of membrane proteins by ER-PM junctions has not been reported on plants, when we examined the distribution of membrane-associated protein AtFlotillin1 (Supplementary Figure S3E), reduced distribution in regions of the membrane displaying either SYT1-YFP or especially the FLS2-StRem1.3 complexes was apparent, as revealed by both maximum intensity projection and orthogonal projection (Supplementary Figures S3B,D) whereas general membrane labeling by FM4-64 was not restricted (Supplementary Figures S3A,C).

### StRem1.3 and Other Peripheral Membrane Proteins Can Replace the C-Terminus of SYT1 in ER-PM Tethering

**Figure 3:**
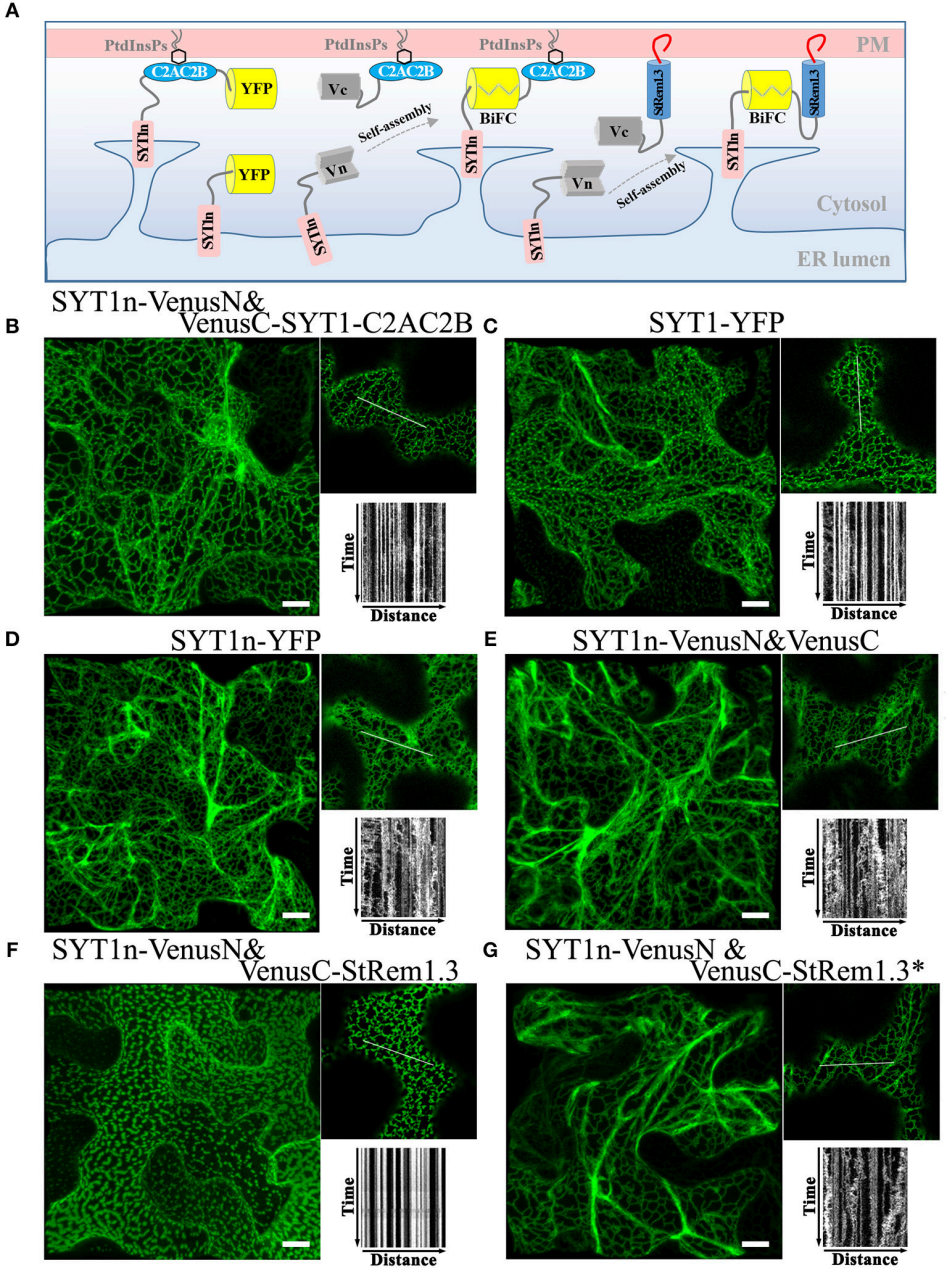
A C-terminal peripheral PM-binding domain is required for ER-PM tethering by *Arabidopsis* SYT1 in *N. benthamiana* leaf cortical cells. **(A)** Explanatory schematic of reconstitution of SYT1 ER-PM tethering using BiFC complexes. **(B,C)** Distribution and kymograph analysis of full length SYT1 or SYT1 reconstituted using Venus BiFC complexes. **(D,E)** Distribution and kymograph analysis of SYT1 lacking the C-terminal peripheral PM-binding domain C2AC2B labeled by either full-length YFP or a free Venus BiFC complex. **(F,G)** Distribution and kymograph analysis of SYT1n fused to WT-type StRem1.3 or PM-non-binding mutant StRem1.3^*^ via Venus BiFC complexes. All kymographs were created as described in Figure 2. All scale bars represent 10 μm.

The ER-PM tethering protein SYT1 contains an N-terminal ER transmembrane domain (SYT1n) and a C-terminal peripheral PM-binding C2AC2B domain (Prinz, 2014; Figure 3A). As shown in Figure 3B, formation of a Venus BiFC complex was sufficient to reconstitute the membrane tethering function of the separated SYT1 N- and C-terminal domains. The complex showed the same distribution and stability as intact SYT1 (Figure 3C, Supplementary Figure S4). Removal of the C-terminal C2AC2B domain of SYT1 resulted in a dynamic net-like distribution, whether the SYT1 N-terminus was labeled with full length YFP (Figure 3D, Supplementary Figure S4) or a Venus BiFC complex (Figure 3E). This dynamic distribution co-localized with the ER marker SP-tagRFP-HDEL (Supplementary Figure S4). However, adding StRem1.3 to the C-terminus of SYT1n via a BiFC complex was sufficient to restore the stable ER-PM site distribution (Figure 3F) and could also partially stabilize the distribution of co-expressed SP-tagRFP-HDEL (Supplementary Figure S4). When StRem1.3 was replaced by the mutant StRem1.3^*^, the ability to reconstitute ER-PM tethering with SYT1n was abolished (Figure 3G). In addition, to reduce the possibility that tethering is an artifact of over-expression in the *N. benthamiana* transient system, we examined different times of expression. As shown in Supplementary Figure S5, protein fluorescence was barely visible at 18 hours post infiltration (hpi). At the earliest time point when there was sufficient expression for reliable fluorescence imaging (36 hpi), the distribution patterns were the same as at the later time point of 72 hpi, both for SYT1n-VenusN + VenusC-SYT1-C2AC2B and for SYT1n-VenusN + VenusC-StRem1.3.

To test if other peripheral membrane proteins could also participate in ER-PM tethering, we replaced the C2AC2B domain of SYT1 with the well-studied receptor-like cytoplasmic kinases (RLCKs), BIK1 (Lu et al., 2010), PBS1 (Qi et al., 2014), or CPK21 (Asai et al., 2013); these three proteins are targeted to the PM by either by N-terminal myristoylation, palmitoylation or both (Supplementary Figure S6). Co-expression of BIK1-VenusN + SYT1n-VenusC, PBS1-VenusN + SYT1n-VenusC, and CPK21-VenusN + SYT1n-VenusC all produced stable puncta-like distributions resembling ER-PM tethering (Supplementary Figure S6), which was further confirmed by co-localization analysis using SYT1 fused with tagRFP (Supplementary Figure S6).

### Integral Membrane Proteins Can Contribute ER Anchoring to Produce ER-PM Tethering

**Figure 4:**
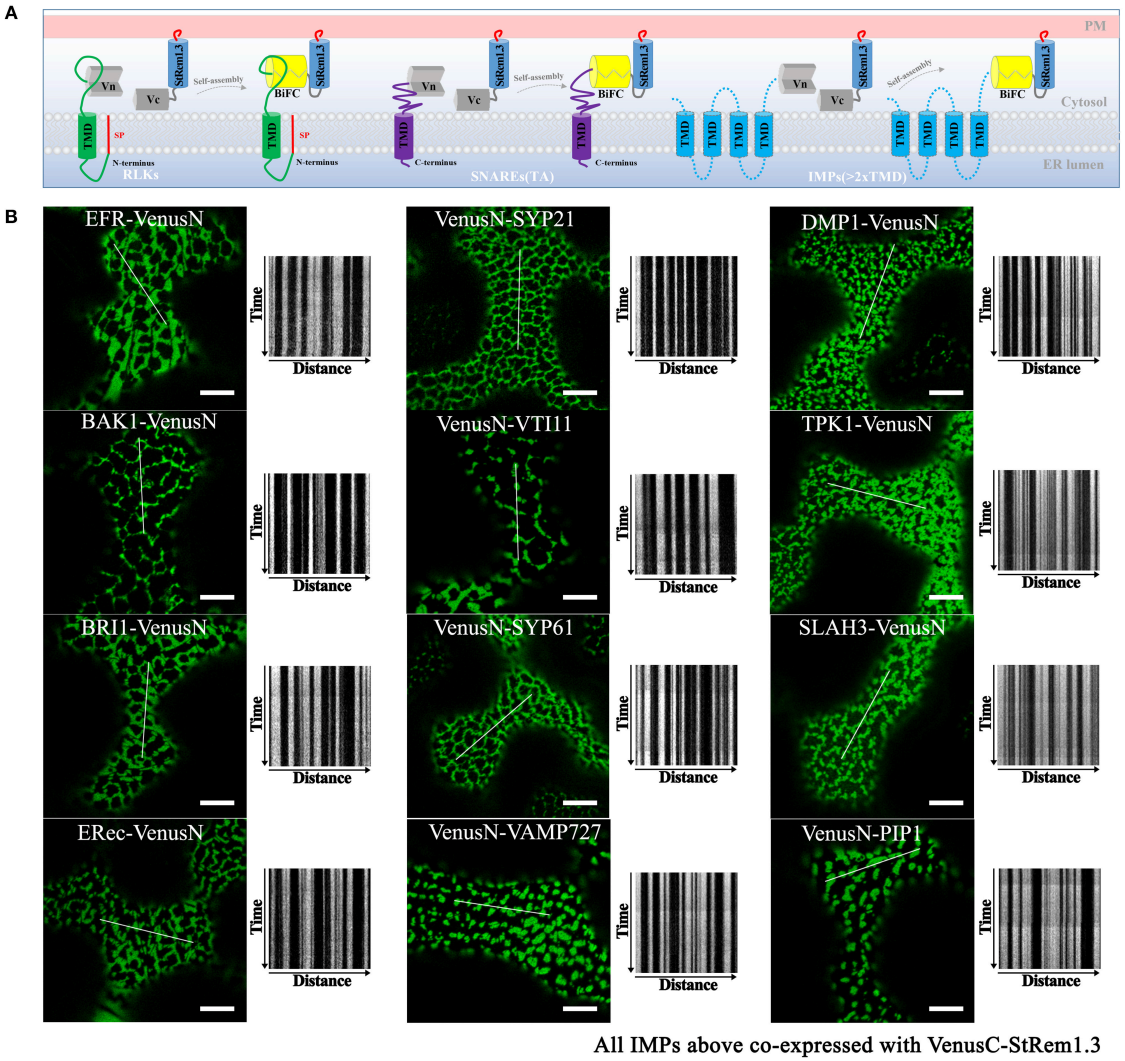
Integral membrane proteins co-expressed with StRem1.3 in Venus BiFC complexes produce EM-PM tethering in *N. benthamiana* leaf cortical cells. **(A)** Explanatory schematic of representative classes of IMPs tested: IMPs with a cleavable N-terminal signal peptide and single-pass TMD; C-terminally anchored IMPs with single-pass TMD (tail-anchored SNARE proteins); IMPs with multi-pass TMDs. **(B)** Localization and kymograph analysis of Venus BiFC complexes formed with each IMP and StRem1.3. All kymographs were created as described in Figure 2. All scale bars represent 10 μm.

Integral membrane proteins (IMPs) such as FLS2 are synthesized on the ER, with the N-terminal domain in the lumen of the ER and the C-terminal domain in the cytoplasm (Walter and Johnson, 1994; Goder and Spiess, 2001). We hypothesized that the reason that FLS2 could participate in tethering-like complexes was because the formation of the FLS2-StRem1.3 complex trapped FLS2 in the ER, with its C-terminal-attached VenusN or VenusC fragment in the cytoplasm, bound to the StRem1.3 component (Figure 4A). As demonstrated above (Figure 1), StRem1.3 can contribute the PM-binding required for ER-PM tethering, while the StREM1.3^*^ mutant that lacks PM-binding cannot (Supplementary Figure S1B). Thus we inferred that binding of the StRem1.3 component to the PM could trap the FLS component in the ER.

To address whether other IMPs could also form this ER-PM tethering structure with StRem1.3 through BiFC self-assembly, we selected several well-studied plasma membrane RLKs which share similar structural characteristics with FLS2 and also have similar localization patterns (Supplementary Figure S7). RLKs are integrated into the ER through the co-translational translocation machinery. We selected EF-Tu receptor (EFR) (Zipfel et al., 2006), brassinosteroid-associated kinase (BAK1) (Heese et al., 2007), BRI1 (Russinova et al., 2004), and ERECTA receptor (ERec) (Bemis et al., 2013). When co-expressed with StRem1.3 in BiFC complexes, all these RLKs produced stable distribution patterns consistent with ER-PM tethering (Figure 4B).

Next we tested IMPs that insert into the ER membrane post-translationally. For this purpose we selected tail-anchored (TA) proteins. These proteins lack an N-terminal signal peptide but contain a single transmembrane domain (TMD) which resides so close to the C terminus that it cannot be recognized by the signal recognition particle (SRP) (Rapoport, 2007; Hegde and Keenan, 2011). We selected a set of *Arabidopsis* TA SNARE proteins, namely SYP21 (Qa-SNARE) (Foresti et al., 2006), VTI11 (Qb-SNARE) (Sanmartín et al., 2007), SYP61 (Qc-SNARE) (Hachez et al., 2014), and VAMP727 (R-SNARE) (Ebine et al., 2008). In these proteins, the C-terminal TMD determines their localization in vesicles of the secretory and endocytic pathways (Supplementary Figure S7). In each case, co-expression of these TA SNARE proteins with StRem1.3 in BiFC complexes resulted in a stable distribution consistent with ER-PM tethering (Figure 4B).

We also tested several IMPs which span the membrane bilayer more than once, that reside on the PM, endosomal membranes, and vacuolar membrane (Supplementary Figure S7). We tested *Arabidopsis* DUF679 membrane protein (AtDMP1) (Kasaras et al., 2012), tonoplast potassium channel protein AtTPK1 (Maîtrejean et al., 2011), slow anion channel 1 (SLAC) homolog SLAH3 (Demir et al., 2013), and intracellular aquaporin PIP1 (Wudick et al., 2009). As shown in Figure 4B, a pattern consistent with ER-PM tethering was observed when each of the multi-pass IMPs was co-expressed with StRem1.3 in BiFC complexes. Together, the above results suggest that patterns consistent with ER-PM tethering were produced with multiple types of IMPs.

Additionally, observations of FLS2, SYP21, or AtDMP1 BiFC complexes formed with StRem1.3 after different times of expression or in cells with different expression levels suggested that the formation of tethering complexes was not an artifact of over-expression (Supplementary Figure S8). While the complexes were barely visible at 18 hpi, the patterns at 36 hpi (the earliest time when visualization was reliable) were almost identical as at 72 hpi. Cells with low levels of BiFC complex formation generally exhibited discrete punctae resembling EPCSs, while in cells with higher levels, the punctae were larger and in some cases merged to form network patterns (Supplementary Figure S8).

In contrast to the IMPs, we did not observe distributions consistent with ER-PM tethering when peripheral membrane proteins were paired with StRem1.3 in BIFC complexes. BIK1-VenusN, PBS1-VenusN, and CPK21-VenusN co-expressed with VenusC-StRem1.3, produced only homogeneously distributed BiFC signals on the PM (Supplementary Figure S9). Similar results were also obtained with *Arabidopsis* SNAP33 (Kargul et al., 2001; Jahn and Scheller, 2006) which has been recognized as a membrane targeted Qbc-SNARE protein lacking a TMD (Supplementary Figure S9). Collectively, these results imply at least one IMP is required in the BiFC complex to produce ER-PM tethering.

### PtdIns(4)P- and PtdIns(4,5)P_2_-Binding Proteins Could Replace Peripheral Membrane-Binding Proteins in BiFC Complexes to Produce ER-PM Tethering

**Figure 5:**
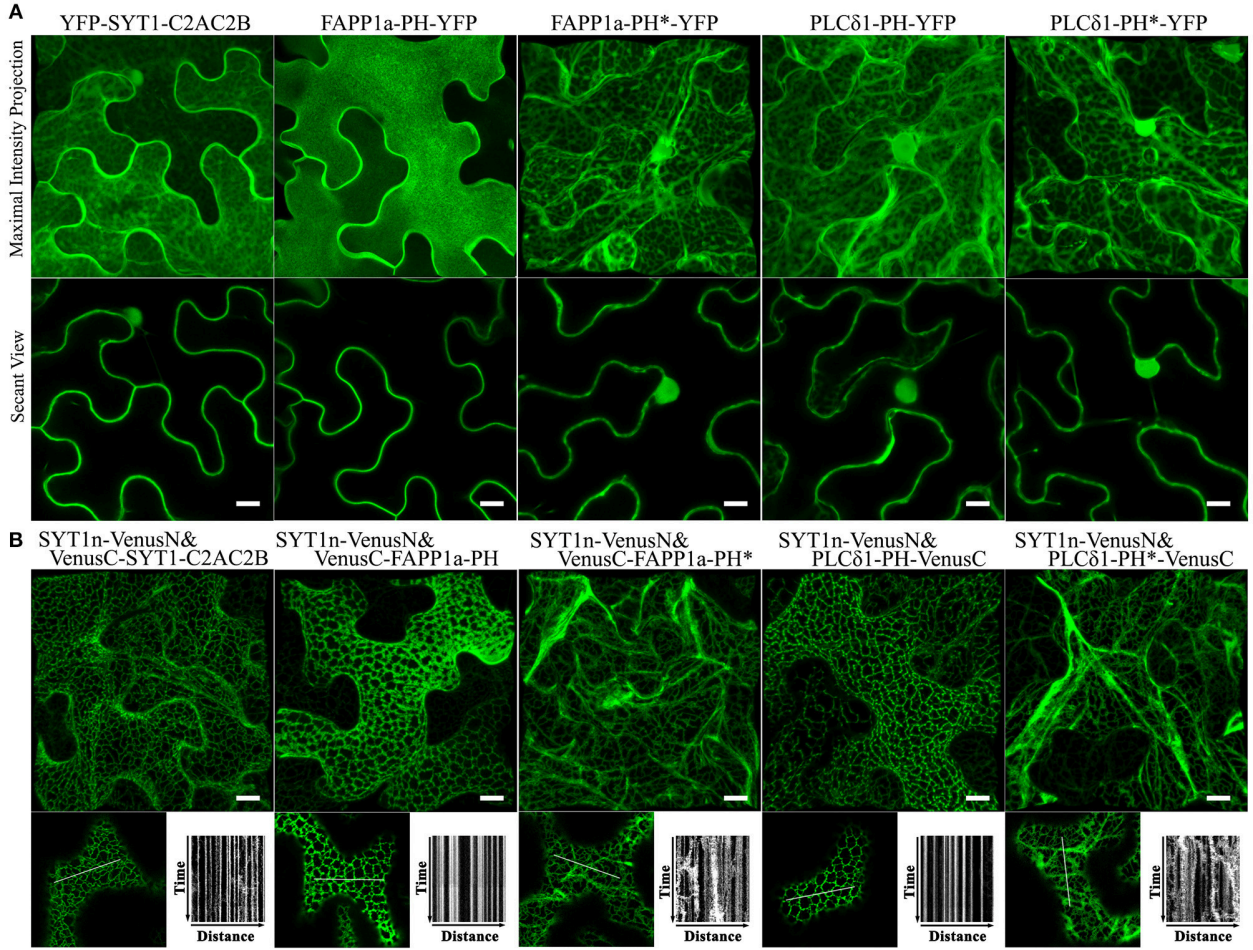
Lipid binding proteins co-expressed with the SYT1 N-terminal domain in BiFC complexes produce EM-PM tethering in *N. benthamiana* leaf cortical cells. **(A)** Subcellular localization of YFP-fused lipid-binding domains SYT1-C2AC2B, FAPP1a-PH, and PLCδ1-PH, together with lipid-non-binding mutants FAPP1a-PH^*^ and PLCδ1-PH^*^. **(B)** Localization and kymograph analysis of BiFC complexes formed from lipid binding domains and mutants fused with VenusC and co-expressed with SYT1n-VenusN. All kymographs were created as described in Figure 2. All scale bars represent 10 μm.

SYT1 normally binds acidic phospholipids in the PM via a C2 domain (Pérez-Sancho et al., 2015; Figure 3). Pleckstrin homology (PH) domains are a large family of phosphoinositide-binding proteins with a broad range of specificities (Lemmon, 2008). Fluorescent protein-tagged PH domain proteins have been used in plants and other organisms to detect PtdIns(4)P and PtdIns(4,5)P_2_ In plants, the PM has been identified as a pool for both PtdIns(4)P and PtdIns(4,5)P_2_ (Van Leeuwen et al., 2007; Vermeer et al., 2009). We therefore tested whether PtdIns(4)P and PtdIns(4,5)P_2_ binding PH domain proteins could replace the C2 domains of SYT1 for ER-PM tethering. We used the PH domains of the PtdIns(4)P-binding protein FAPP1 (Dowler et al., 2000), and of the PtdIns(4,5)P_2_ binding protein PLC-delta 1 (Yagisawa et al., 1998); both have been well-characterized in animal cell systems and have been used in plants previously (Van Leeuwen et al., 2007; Vermeer et al., 2009; Simon et al., 2016). In the case of FAPP1, we used a mutant of FAPP1 (E50A, H54A; hereafter named FAPP1a) that no longer binds the golgi protein ARF1 (Simon et al., 2016). As a negative binding control, we designed site-directed mutants of each biosensor lacking lipid binding based on previous reports (Yagisawa et al., 1998; He et al., 2011). To begin with, we performed subcellular localization assays on YFP-tagged SYT1-C2AC2B, FAPP1a-PH, FAPP1a-PH^*^, PLCδ1-PH, and PLCδ1-PH^*^ (^*^indicates the mutants). Similar to the results previously observed for C2AC2B-GFP (Pérez-Sancho et al., 2015), YFP-PH^FAPP1−E50A−H54A^ (Simon et al., 2016), and YFP-PH_PLCδ1_ (Van Leeuwen et al., 2007), we observed that FAPP1a-PH-YFP was more strictly localized at the PM than YFP-SYT1-C2AC2B and PLCδ1-PH-YFP, which additionally displayed cytosolic and nuclear localizations (Figure 5A). In contrast, both the FAPP1a-PH^*^ and PLCδ1-PH^*^ mutants showed diffuse patterns of cytosolic localization (Figure 5A). When co-expressed with SYT1n-VenusN, all three of VenusC-SYT1-C2AC2B, VenusC-FAPP1a-PH, and PLCδ1-PH-VenusC showed patterns consistent with ER-PM tethering (Figure 5B), while the mutant PH domains produced only dynamic patterns associated with ER localization (Figure 5B). In contrast, when the PtdIns(3)P-binding protein domains, VAM7-PX (Cheever et al., 2001) or Hrs-2xFYVE (Vermeer et al., 2006), were provided as potential PM-binding proteins, only dynamic ER localization patterns were observed (Supplementary Figure S10), comparable to the FAPP1a-PH^*^ and PLCδ1-PH^*^ mutants. Furthermore, PtdIns(3)P-non-binding mutants of VAM7-PX and Hrs-2xFYVE, which were designed according to previously identified binding sites (Kutateladze and Overduin, 2001; Lee et al., 2006; Pankiv et al., 2010), produced distribution patterns (Supplementary Figure S10) comparable to their wild type counterparts. Interestingly, the SYTn-PLCδ1-PH BiFC complexes produced a very fine network distribution, with well-defined puncta, that was closely similar to the pattern produced by SYTn-SYT1-C2AC2B BiFC complexes. In contrast, the SYTn-FAPP1a-PH BiFC complexes exhibited a thicker network with very abundant puncta, comparable to the patterns exhibited SYT1n-StRem1.3 BiFC complexes. We speculate that this difference may be associated with the stronger PM localization exhibited by FAPP1a-PH and StRem1.3 (Figure 5A, Supplementary Figure S1). In conclusion, our data suggest that PtdIns(4)P- and PtdIns(4,5)P_2_-binding proteins, but not PtdIns(3)P-binding proteins, could contribute the PM-targeting needed for ER-PM tethering.

## Discussion

**Figure 6:**
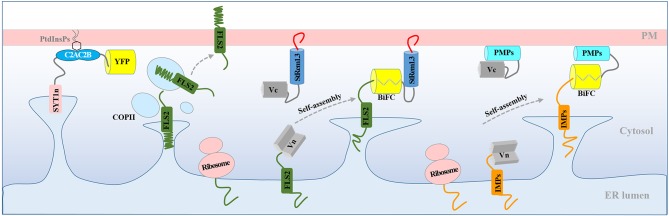
Model for the production of ER-PM tethering complexes via BiFC. Normally, newly synthesized RLK protein FLS2 is targeted to the PM through the ER and transported to the PM via the coat protein complex II (COPII) system. Co-expression of FLS2 and StRem1.3 in BiFC constructs results in rapid spontaneous formation of Venus BiFC complexes tethered to the PM. PM tethering blocks ER-anchored FLS2 from delivery to the PM, resulting in artificial PM-ER complexes. Artificial ER-PM tethering could be created by pairing any ER-transiting integral membrane protein (IMP) with any peripheral membrane protein (PMP) in a BiFC complex.

In this study, we observed that BiFC complexes containing both the RLK, FLS2, and the membrane-associated remorin protein, StRem1.3, exhibited a range of heterogeneous distribution patterns closely resembling those produced by over-expression of the *Arabidopsis* ER-PM tether protein, SYT1 (Figures 1, 2). Indeed, co-expression of the FLS2-StRem1.3 BiFC complexes with SYT1 produced fully overlapping distributions (Figure 2), suggesting that the FLS2-StRem1.3 BiFC complexes might act as artificial ER-PM tethering proteins (Figure 6). Since the gap between the ER and the PM is in the 15–20 nm range (McFarlane et al., 2017) and therefore is too small to be resolved by light microscopy (diffraction limit), we used the stability of tethering sites to distinguish them from the more dynamic free ER networks, using kymographs. We note also that since we did not use electron microscopic observations, we cannot rule out that the stable puncta labeled by SYT1-FP are not true contact sites. The co-location of SYT1 with the artificial tethering sites suggests that the artificial sites might develop into true contact sites. In future, co-expressing the tethering constructs with NET3C might provide additional information about the relationship of the tethering sites we observe with bona fide contact sites (Wang et al., 2014).

We showed that StRem1.3 and also the lipid-conjugated peripheral membrane proteins BIK1, PBS1, and CPK21, could replace the PM-binding C2 domain of SYT1 (Figure 3, Supplementary Figure S6) to produce ER-PM tethering. We showed that the phosphoinositide-binding PH domains from FAPP1 and PLCδ1 could also functionally replace the C2 domain of SYT1 (Figure 5). Finally, we showed that a wide variety of IMPs that transit the ER, including 5 RLKs, 4 tail-anchored proteins, and 4 multi-transmembrane domain proteins, could provide the EM-anchoring function when paired with StRem1.3 as the PM-anchoring protein (Figure 4). In contrast, peripheral membrane proteins that do not transit the ER could not provide the ER-anchoring function (Supplementary Figure S9). On the basis of these observations, we have concluded that FLS2-StRem1.3 BiFC complexes may in fact act as artificial ER-PM tethering proteins. More generally, our model is that any ER-transiting IMP paired with a peripheral membrane protein, either in a BiFC complex or through a direct linkage, may act as an artificial ER-PM tethering protein (Figure 6). In this model, the IMP must transit through the ER, either co-translationally or post-translationally (Walter and Johnson, 1994; Goder and Spiess, 2001). Furthermore, the binding of the peripheral membrane protein to the PM should be sufficiently strong to trap the IMP in the ER, and prevent the completion of the IMP's transit to its final membrane destination. Conversely, the peripheral membrane protein should be synthesized in the cytoplasm and then be targeted to the PM post-translationally, without entering the ER, either via conjugation to a lipid, binding to a PM lipid, or via insertion of a hydrophobic loop or helix (Vögler et al., 2008; Pu et al., 2010; Resh, 2013; Stillwell, 2016).

In plants, several studies have reported observing heterogeneous distributions of BiFC complexes that combine IMPs with peripheral membrane proteins. However, none of these studies have considered the possibility that the distribution patterns observed may have arisen as a result of the formation of artificial ER-PM tethering proteins. For example, Jarsch et al. (2014) showed that whereas the FP-tagged RLK MtNFR1 and remorin MtSYMREM1 were uniformly distributed across the PM when individually expressed in *N. benthamiana* leaf epidermal cells, when the two were co-expressed in a BiFC complex, the fluorescent signal was exclusively observed in distinct, immobile puncta. Similarly, Demir et al. (2013) observed that BiFC complexes comprised of *Arabidopsis* SLAH3 (an IMP) and CPK21 (a PMP) localized to distinct PM puncta. Likewise, Bücherl et al. (2017) expressed the following RLK-PMP proteins pairs in BiFC complexes and observed the formation of distinct puncta on the PM: FLS2-BSK1, BRI1-BSK1, FLS2-BIK1, and BRI1-BIK1. Our data suggest that it is necessary to re-evaluate the applicability of BiFC assays for plant membrane studies, and the validity of published studies that used this approach (e.g., Demir et al., 2013; Jarsch et al., 2014; Bücherl et al., 2017) should be re-visited.

Unambiguously determining PM localization in plant cells is challenging. In comparison to mammalian cells, many plant cells contain a large central vacuole that takes up most of the cell volume, resulting in the cytoplasm and organelles being constrained into the periphery of the cell and appressed to the PM. Several methods have been commonly used to distinguish the PM from the vacuolar membrane (tonoplast), including plasmolysis (Speth et al., 2009) and osmolysis (Serna, 2005). However, these methods may be confounded by the presence of the tonoplast or of overexpression artifacts. For example, we observed that some weakly binding PMPs, e.g., SNAP33 (Supplementary Figure S9), SYT1-C2AC2B (Figure 5), and PLCδ1-PH (Figure 5), show substantial cytoplasmic localization when they are over-expressed as FP fusions. The ability of PMPs to form ER-PM tethering complexes may in some circumstances aid in distinguishing between cytosolic and membrane proteins. For example, there is currently not a strong consensus as to the localization of PtdIns(4,5)P_2_ in plant cells (Delage et al., 2012). Although PtdIns(4,5)P_2_ has been well established as a PM lipid in animal cells (Hammond et al., 2012), evidence for the same localization in plant cells has been ambiguous (Van Leeuwen et al., 2007). Our observations that PLCδ1-PH is effective in forming ER-PM tethering complexes with SYT1n suggests that PtdIns(4,5)P2 is indeed located in the plant PM (but does not rule out other locations as well). In contrast, our negative tethering results with PtdIns(3)P-binding proteins suggest that this lipid does not reside on the cytoplasmic face of the PM.

Given the ability of IMPs to act as the ER-anchor in artificial ER-PM tethering complexes, such complexes could possibly be used to investigate whether a protein may be an IMP or not, as suggested previously by Zamyatnin et al. (2006). Bioinformatic analysis has been increasingly used to predict the identity and topology of IMPs. However, these algorithms are not fully accurate. For example, two commonly used programs TMHMM (Krogh et al., 2001) and Protter (Omasits et al., 2014) can differ in their predictions. Artificial ER-PM tethering complexes could be used to test such predictions. As one example from this work, Protter and TMHMM both predicted a weak TMD in CPK21 whereas all other members of the CDPK family are targeted to the PM by myristoylation and palmitoylation (Speth et al., 2009; Asai et al., 2013; Schulz et al., 2013), suggesting that the bioinformatic prediction may be incorrect. In fact, we observed that CPK21-SYT1n BiFC complexes exhibited tethering (Supplementary Figure S6) but CPK21-StRem1.3 BiFC complexes did not (Supplementary Figure S9), confirming CPK21 as a PMP, not an IMP. Further examples will be needed to determine if this approach is generally useful.

Genetically designed chimeric proteins have been successfully developed to manipulate tethering of the ER to the PM or to other organelles, and to study cellular processes involving tethering proteins (Kornmann et al., 2009; Chang et al., 2013; Bockler and Westermann, 2014; Poteser et al., 2016; Lee et al., 2019). For example, a chimeric protein named MAPPER was used as a constitutive ER-PM tethering marker to investigate the molecular mechanism for dynamic regulation of ER-PM tethering during Ca^2+^ signaling in live mammalian cells; MAPPER is derived from the human ER-PM tether STIM1 and contains minimal ER and PM-targeting motifs, linked by a fluorescent protein (Chang et al., 2013; Poteser et al., 2016). More recently, MAPPER has also been used as a non-regulated ER-PM tethering marker to study the response of *Arabidopsis* SYT1 on the regulation of ER-PM connectivity under ionic stress (Lee et al., 2019). Additionally, ChiMERA, a synthetic ER-Mitochondria tether consisting of GFP fused to the mitochondrial membrane anchored TMD motif of Tom70 and an ER tail-anchor motif from Ubc6, was used to restore mitochondria-ER contacts in yeast mutants (Kornmann et al., 2009; Bockler and Westermann, 2014). Therefore, our work here not only suggests a potential way to develop fluorescent molecular markers for EPCSs, but also suggests a molecular tool to manipulate tethering of the ER to the PM, or even to other membrane organelles in plants. One can imagine, for example, tethering complexes in which dimerization of the two components is regulated by small molecules and/or light as reported previously (Karginov et al., 2011; Guntas et al., 2015).

In summary, we have deployed an extensive toolset of plant membrane marker proteins and mutant controls (summary in Table 1, Supplementary Table S1) to characterize the artificial ER-PM tethering that may result from spontaneous reassembly of fragmented fluorescent proteins during co-localization studies. These results complement our findings that a similar phenomenon can produce tethering of multi-vesicular bodies and the tonoplast to the PM (Tao et al., 2019). Our results indicate the possibility of new tools for deliberately manipulating ER-PM tethering, while at the same time highlighting a previously unrecognized artifact that may have confounded several published studies.

**Table 1:** Fluorescent marker proteins and mutants used in this study.

**Protein**	**Accession/Tair**	**Region**	**Localization in *N. benthamiana* leaf cortical cells**	**Features**	**References**
**SUBCELLULAR MARKERS**					
FLS2	AT5G46330.1	FL	PM	IMP	Gómez-Gómez and Boller, 2000
StRem1.3	NP_001274989	FL	PM	PMP	Perraki et al., 2012
StRem1.3^*^		FL	Cytoplasm, nuclear	C terminal mutations eliminating PM-binding L179H, A180H, A181H, Y184S, A185S, G187V, A189A, L194S, G195Q, I196Q, F197Q	Perraki et al., 2012
SYT1	AT2G20990	FL	ER-PM tether	IMP	Pérez-Sancho et al., 2015
SYT1n	AT2G20990	1–243	ER membrane	N-terminal domain of SYT1	Pérez-Sancho et al., 2015
SYT1-C2AC2B	AT2G20990	244–541	PM (mainly), cytoplasm, nuclear	C-terminal domain of SYT1 binding to a variety of negatively charged phospholipids	Pérez-Sancho et al., 2015
AtFlotillin1	AT5G25250	FL	PM	PMP	Jarsch et al., 2014
BIK1	At2g39660	FL	PM	PMP	Lu et al., 2010
PBS1	AT5G13160	FL	PM	PMP	Qi et al., 2014
CPK21	AT4G04720	FL	PM	PMP	Asai et al., 2013
EFR	AT5G20480	FL	PM	IMP	Zipfel et al., 2006
BAK1	AT4G33430.1	FL	PM, though slight cell death observed	IMP	Heese et al., 2007
BRI1	AT4G39400.1	FL	PM	IMP	Russinova et al., 2004
ERec	AT2G26330.1	FL	PM	IMP	Bemis et al., 2013
SYP21	AT5G16830	FL	MVB, tonoplast	IMP	Foresti et al., 2006
VTI11	AT5G39510	FL	Golgi, MVB, tonoplast	IMP	Sanmartín et al., 2007
SYP61	AT1G28490	FL	TGN/EE	IMP	Hachez et al., 2014
VAMP727	AT3G54300	FL	Endosomal organelles (partially MVBs), tonoplast	IMP	Ebine et al., 2008
SNAP33	AT5G61210	FL	PM (not visible by regular FP-tagged localization analysis), cytoplasm (mainly), nuclear	PMP	Kargul et al., 2001
AtDMP1	AT3G21520.1	FL	MVBs, tonoplast (mainly)	IMP (GFP inserted between 108E and 109P)	Kasaras and Kunze, 2017
AtTPK1	AT5G55630	FL	MVBs, tonoplast (mainly)	IMP	Maîtrejean et al., 2011
PIP1	AT2G36830	FL	Endosomal organelle	IMP	Wudick et al., 2009
FAPP1a-PH	AAG15199	1–99	PM	FAPP1-PH protein containing mutations of the ARF1 binding site: E50A, H54	He et al., 2011
FAPP1a-PH^*^		1–99	Cytoplasm, nuclear	Mutations of PtdIns(4)P binding site K7E, R18A	He et al., 2011
PLCδ1-PH	AAH50382	1–174	PM (not visible by regular FPs-tagged localization analysis), cytoplasm (mainly), nuclear	PtdIns(4, 5)P binding	Yagisawa et al., 1998
PLCδ1-PH^*^		1–174	Cytoplasm, nuclear	Mutations of PtdIns(4,5)P binding site K30A, K32E, R40A	Yagisawa et al., 1998
tagRFP-HDEL			The lumen of endoplasmic reticulum	Includes Ssignal peptide (MGYMCIKISFCVMCVLGLVIVGDVAYA) cloned from soybean (Glycine max) secreted protein PR1a precursor (Accession: NP_001238168)	Matsushima et al., 2002
SLAH3	AT5G24030	FL	PM	IMP	Demir et al., 2013
VAM7-PX	NP_011303	1–134	MVBs, tonoplast	PtdIns(3)P binding	Kale et al., 2010
VAM7-PX^*^		1–134	Cytoplasm, nuclear	Mutations of PtdIns(3)P binding site R40E, S42A	Lee et al., 2006
Hrs-2xFYVE	NP_032270	147–223	MVBs, tonoplast	Tandem repeat of PtdIns(3)P binding domain, linked by QGQGS	Vermeer et al., 2006
Hrs-2xFYVE^*^		147–223	Cytoplasm, nuclear	Mutations of both PtdIns(3)P binding sites R34S, K35S, H36S, H37S, R39S	Kutateladze and Overduin, 2001; Pankiv et al., 2010

* *mutant; ER, endoplasmic reticulum; FL, full length; IMP, integral membrane protein; MVB, multivesicular bodies; PM, plasma membrane; PM-MVB/TP, plasma membrane-multivesicular body/tonoplast tethering sites; PMP, peripheral membrane protein; SP, signal peptide; tagRFP, tag red fluorescent protein; TP, tonoplast; YFP, yellow fluorescent protein*.

## Experimental Procedures

### Plant Materials

*Nicotiana benthamiana* and *A. thaliana* plants were grown in soil (Fafard^®^ 4M Mix). *N. benthamiana* plants were grown in a growth chamber with a 14 h photoperiod at 25°C for 5 weeks before *A. tumefaciens* infiltration assays. *A. thaliana* seeds were sown in soil and left at 4°C for 3 day of cold stratification. Then the seedlings were grown in a growth chamber with a 12 h photoperiod at 20°C for 4 weeks before protoplast isolation.

### Cloning and Construction

FLS2, BAK1, BRI1, ER, BIK1, PBS1, FAPP1-PH, Hrs-2xFYVE, VAM7-PX were sub-cloned from constructs described previously (Kale et al., 2010; Lu et al., 2010; Meng et al., 2015). The SYT1 (AT2G20990.1), AtDMP1 (AT3G21520.1), AtTPK1 (AT5G55630.1), AtPIP1 (AT3G61430.1), SLAH3 (AT5G24030.1), CPK21 (AT4G04720.1), SNAP33 (AT5G61210.1), AtSYP21 (AT5G16830.1), AtVTI11 (AT5G39510.1), AtVAMP727 (AT3G54300.1), AtSYP61 (AT1G28490.1), AtFlotillin1 (AT5G25250) coding regions were amplified from *Arabidopsis* Col-0 cDNA. StRem1.3 (U72489.1), and PLCδ1-PH (BC050382.1) were synthesized by GenScript Corporation. All PCR amplifications were performed by High-Fidelity DNA polymerase (CloneAmpTM HiFi PCR Premix, TaKaRa Bio). All PCR products were individually recombined by In-Fusion^®^ HD Cloning (TaKaRa Bio) into the Gateway vector pDONR207 into which VenusN, YFPn, VenusC, YFPc, or full-length FPs had previously been inserted. The ER marker was generated by using PCR mutagenesis to add a carboxyl-terminal HDEL peptide to tagRFP, then cloning tagRFP-HDEL into a vector (psAEV) that provided a signal peptide. The site-specific mutations of PLCδ1-PH^*^, StRem1.3^*^, FAPP1a-PH^*^, VAM7-PX^*^, and Hrs-2xFYVE^*^ were introduced into their respective pDONR207 constructs using appropriate oligonucleotides in a PCR reaction that amplified the entire vector. A list of primers designed and used are in Supplementary Table S2. By using the Gateway^®^ LR reaction (Thermo Fisher scientific Inc.), all constructs were transferred from their pDONR207 vectors into the destination expression vectors namely pmAEV, which is derived from binary vectors pCAMBIA (Dou et al., 2008) and driven by the Cauliflower Mosaic Virus (CaMV) 35S promoter conferring constitutive high level expression in plant cells. All these plasmid constructs were confirmed by DNA sequencing at the Center for Genome Research and Biocomputing (Oregon State University).

### Transient Expression in *N. benthamiana* Leaves and *A. thaliana* Protoplasts

The procedures to introduce expression vectors into *A. tumefaciens* strain GV3101, and to infiltrate transformed *A. tumefaciens* cells into 5-week-old *N. benthamiana* leaves were carried out as described previously (Lu et al., 2013). *A. tumefaciens* cells were infiltrated at OD_600_ of 0.1 for the expression of the full-length fluorescent protein tagged proteins; for co-expression of BiFC constructs, two *A. tumefaciens* cultures with OD_600_ of 0.2, respectively, were equally mixed together to reach the final OD_600_ at 0.1. All infiltrated *A. tumefaciens* cells were suspended in MES buffer (10 mM MgCl_2_, 10 mM MES pH 5.7, and 100 μM acetosyringone). *N. benthamiana* leaves were imaged at 3 days post infiltration. *A. thaliana* mesophyll protoplasts were prepared from leaves of 4-week-old seedlings, and 10 μg of plasmid DNA were used for each transformation as described (Yoo et al., 2007). Following transformation, protoplasts were suspended overnight in W5 buffer (154 mM NaCl, 125 mM CaCl_2_, 5 mM KCl, 2 mM MES pH 5.7) at 25°C before observation.

### Live-Cell Imaging by Confocal Microscopy and Image Analysis

FM 4-64 (ThermoScientificBio) staining employed a concentration of 10 μM, and was performed as previously described (Günl et al., 2011). All microscopy images were obtained using a ZEISS LSM 780 NLO confocal microscope system equipped with a 458-nm argon laser for CFP (emission wavelength 560–509 nm), a 514-nm argon laser for YFP/Venus (emission wavelength 518–553 nm), and a 561-nm Diode Pumped Solid State (DPSS) laser for tagRFP and FM4-64 (emission wavelength 562–640 nm). For time-lapse imaging, 100 consecutive frames without time intervals (combined speed of about 0.78 fps) were acquired sequentially. The kymograph plots were generated using ImageJ (Version 1.51n, NIH) and the plug-in “KymographBuilder” with a 30 μm segmented line for this measurement. When interpreting the kymographs, close attention was paid to distinguishing the cortical ER from transvacuolar strands, which are also dynamic. All microscopy images were processed using the Zeiss ZEN2 (Blue edition) program.

## Author Contributions

KT and BT conceived and planned the experiments and wrote the manuscript. KT performed experiments and analyzed the data. JW and FA were involved in performing experiments.

### Conflict of Interest Statement

The authors declare that the research was conducted in the absence of any commercial or financial relationships that could be construed as a potential conflict of interest.

## Supplementary Material

Supplementary Table S1 Amino acid sequences and other details of protein fusions used in this study.

Supplementary Table S2 Primers designed and used for vector constructions.

Supplementary Movie S1 Dynamics of ER networks labeled by SP-tagRFP-HDEL.

Supplementary Movie S2 Dynamics of heterogeneous patterns produced by FLS-StRem1.3 BiFC complexes.

Supplementary Figure S1 Efficient spontaneous reassembly of the two fragments of Venus into BiFC complexes with membrane proteins in *N. benthamiana* leaf cortical cells. **(A)** PM-localized FLS2 and StRem1.3 BiFC complexes formed with free Venus fragments. **(B)** Distribution of BiFC complexes produced by FLS2-VenusN co-expressed with StRem1.3^*^. **(C)** FLS2, StRem1.3, and its PM-targeting mutant StRem1.3^*^ individually fused with full-length YFP. Scale bars represent 10 μm.

Supplementary Figure S2 Heterogeneous distribution patterns observed for BiFC complexes produced in *N. benthamiana* leaf cortical cells or *Arabidopsis* mesophyll protoplasts. **(A)** PM-localized FLS2 and StRem1.3 BiFC complexes formed with free YFP fragments. Co-expression of FLS2-YFPn and YFPc-StRem1.3 produced a heterogeneous distribution of the BiFC signal similar to that for Venus BiFC shown in Figure 1. **(B)** Transiently expressed in *Arabidopsis* mesophyll protoplasts, FLS2-StRem1.3 BiFC complexes exhibited heterogeneous discrete patches, different from either plasma membrane-localized FLS2-YFP or YFP-StRem1.3, or cytoplasm-localized YFP. Red fluorescence represents chloroplast autofluorescence. BF, bright field. Scale bars in **(A,B)** represent 10 μm.

Supplementary Figure S3 Orthogonal imaging of SYT1 and FLS2-StRem1.3 BiFC complexes in *N. benthamiana* leaf cortical cells, relative to AtFlotillin1 and FM4-64. **(A)** The *Arabidopsis* tethering protein SYT1 tightly associates with the plasma membrane visualized by FM4-64. **(B)** Regions of the plasma membrane associated with SYT1 show reduced presence of membrane protein AtFlotillin1 fused to tagRFP. **(C)** FLS2-StRem1.3 BiFC complexes tightly associate with the plasma membrane, visualized by FM4-64. **(D)** Regions of the plasma membrane associated with FLS2-StRem1.3 BiFC complexes show reduced presence of AtFlotillin1-tagRFP. **(E)** Subcellular localization of AtFlotillin-1-tagRFP when expressed alone. Scale bars represent 10 μm.

Supplementary Figure S4 Distribution and kymograph analysis of SYT1, SYT1n, and SYT1-StRem1.3 BiFC complexes co-expressed with ER marker SP-tagRFP-HDEL in *N. benthamiana* leaf cortical cells. **(A)** SYT1-YFP co-expressed with SP-tagRFP-HDEL. Arrowheads highlight bright puncta resembling ER-PM contact sites that stabilize the dynamic ER. **(B)** SYT1n-YFP co-expressed with SP-tagRFP-HDEL, showing coordinated dynamic mobility. **(C)** FLS2-StRem1.3 BiFC complexes co-expressed with SP-tagRFP-HDEL, showing stabilization of dynamic mobility. Kymographs produced as in Figure 2. Scale bars represent 10 μm.

Supplementary Figure S5 Effect of expression time on the distribution of fluorescently tagged full length SYT1, C-terminal C2AC2B domain of SYT1, N-terminal transmembrane domain SYT1n, and BiFC complexes SYT1n&SYT1-C2AC2B, and SYT1n&StRem1.3 in *N. benthamiana* leaf cortical cells. Hpi = hours since *Agrobacterium* infiltration. Scale bars in black color represent 50 μm, and the white scale bars represent 20 μm.

Supplementary Figure S6 Co-expression of peripheral membrane proteins and SYTn in BiFC complexes in *N. benthamiana* leaf cortical cells results in ER-PM tethering. **(A)** Distribution of peripheral membrane proteins BIK1, PBS1, and CPK21 fused with YFP. **(B)** Distribution and kymograph analysis of BiFC complexes produced by SYT1n-VenusC co-expressed with BIK1-VenusN, PBS1-VenusN, or CPK21-VenusN. **(C)** Puncta observed in BiFC complexes produced by BIK1 & SYT1n, PBS1 & SYT1n, and CPK21 & SYT1n co-localized with wild type SYT1-tagRFP. Kymographs produced as in Figure 2. Scale bars represent 10 μm.

Supplementary Figure S7 Membrane distributions of integral membrane proteins fused to YFP and transiently expressed in *N. benthamiana* leaf cortical cells. **(A)** Subcellular localization of PM IMPs with a cleavable N-terminal signal peptide and single-pass TMD. **(B)** Subcellular localization of tail-anchored SNARE proteins. Qa-SNARE SYP21 and R-SNARE VAMP727 are localized to endosomal vesicles; Qb-SNARE VTI11 and Qc-SNARE SYP61 are localized to the Golgi. **(C)** Subcellular localization of IMPs with multi-pass TMDs. AtDMP1 and AtTPK1 are localized to the vacuolar membrane (tonoplast); SLAH3 is localized on the PM; PIP1 is localized on endosomal membranes. Scale bars in highlighted box represent 5 μm, and all others represent 10 μm.

Supplementary Figure S8 Effect of expression time on the distribution of BiFC complexes FLS2-VenusN + VenusC-StRem1.3, VenusN-SYP21 + VenusC-StRem1.3, and DMP1-VenusN + VenusC-StRem1.3 in *N. benthamiana* leaf cortical cells. Scale bars in black color represent 50 μm, and the white scale bars represent 20 μm. Hpi = hours since *Agrobacterium* infiltration.

Supplementary Figure S9 Co-expression of peripheral membrane proteins and StRem1.3 in *N. benthamiana* leaf cortical cells does not result in ER-PM tethering by BiFC complexes. **(A)** Homogenously distributed fluorescent signal observed in BiFC complexes produced by BIK1 & StRem1.3, PBS1 & StRem1.3, CPK21 & StRem1.3, and SNAP33 & StRem1.3. **(B)** The subcellular localization of Qbc-SNARE protein SNAP33 that lacks a TMD. Scale bars represent 10 μm.

Supplementary Figure S10 Co-expression of PtdIns(3)P binding proteins with the SYT1 N-terminal domain in BiFC complexes in *N. benthamiana* leaf cortical cells does not result in ER-PM tethering. Hrs-2xFYVE, and Vam7-PX proteins are PtdIns(3)P binding proteins while Hrs-2xFYVE^*^ and Vam7-PX^*^ are PtdIns(3)P-non-binding mutants. Kymographs produced as in Figure 2. Scale bars represent 10 μm.
