# Supplementary figures and images for: Manipulating Endoplasmic Reticulum-Plasma Membrane Tethering in Plants Through Fluorescent Protein Complementation
Source: Front Plant Sci. 2019 May 22;10:635. doi: 10.3389/fpls.2019.00635 (PMC6547045; doi:10.3389/fpls.2019.00635)

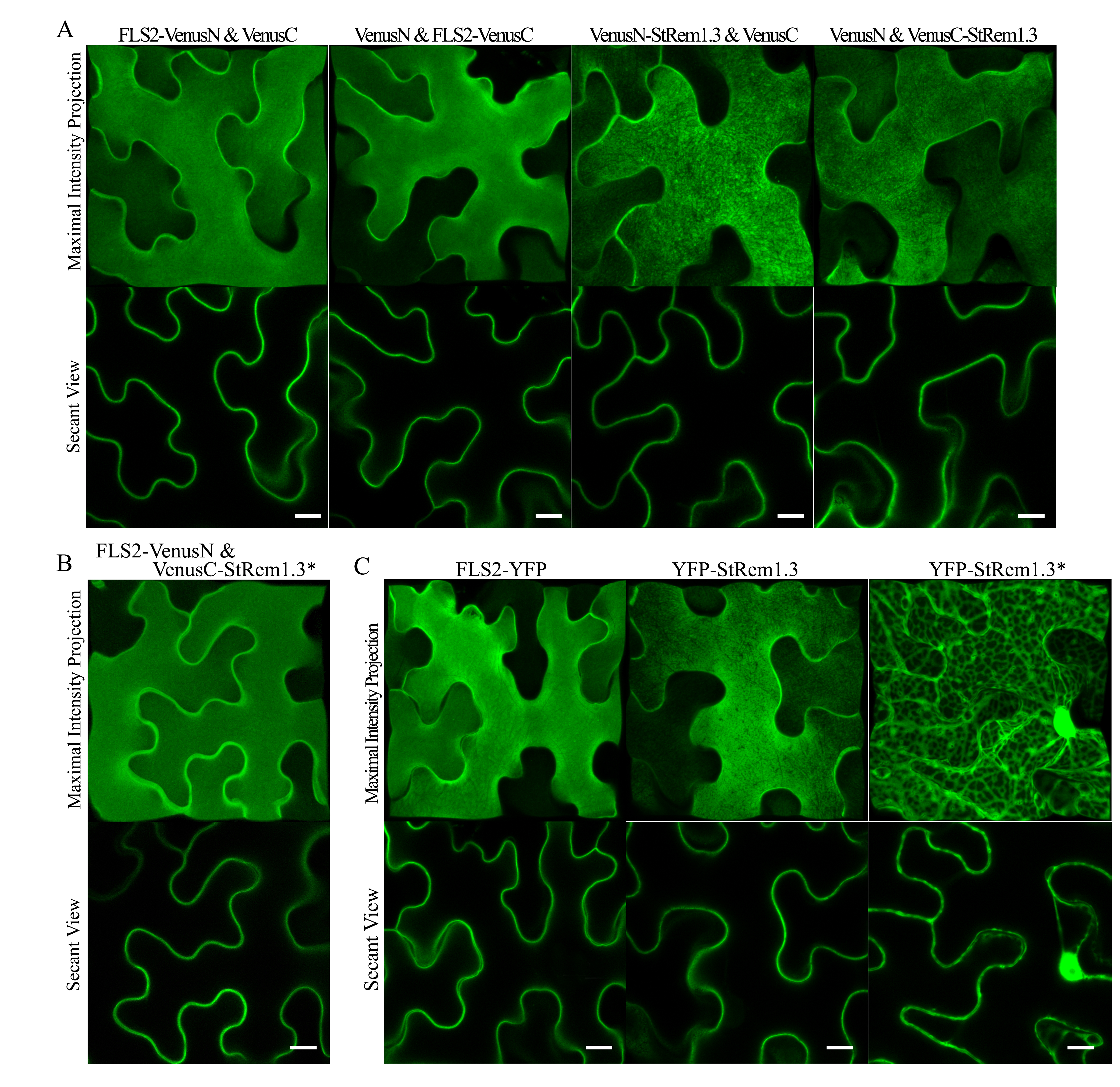

Supplement: Supplementary Figure S1 — Efficient spontaneous reassembly of the two fragments of Venus into BiFC complexes with membrane proteins in N. benthamiana leaf cortical cells. (A) PM-localized FLS2 and StRem1.3 BiFC complexes formed with free Venus fragments. (B) Distribution of BiFC complexes produced by FLS2-VenusN co-expressed with StRem1.3*. (C) FLS2, StRem1.3, and its PM-targeting mutant StRem1.3* individually fused with full-length YFP. Scale bars represent 10 μm. [file Image_1.JPEG]

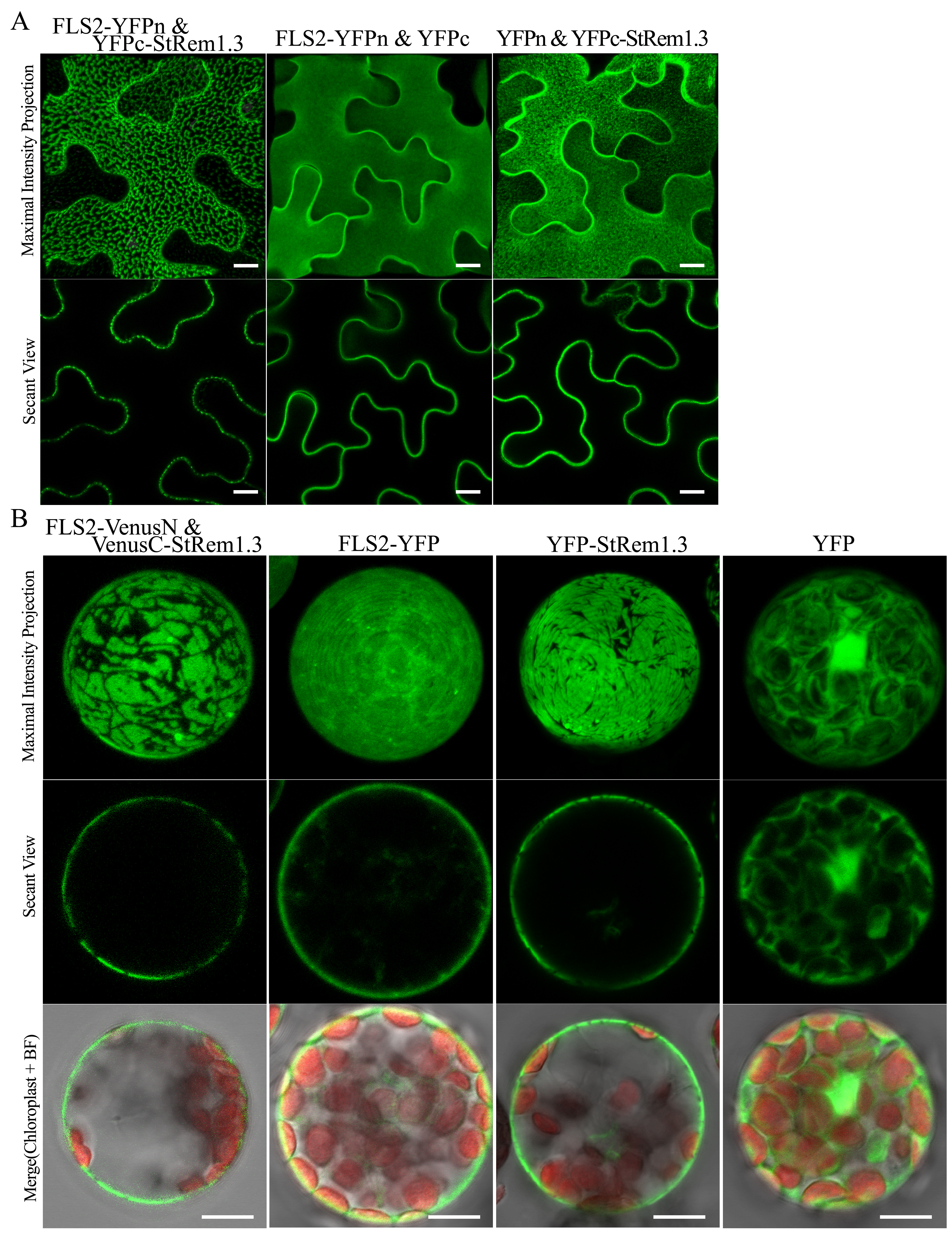

Supplement: Supplementary Figure S2 — Heterogeneous distribution patterns observed for BiFC complexes produced in N. benthamiana leaf cortical cells or Arabidopsis mesophyll protoplasts. (A) PM-localized FLS2 and StRem1.3 BiFC complexes formed with free YFP fragments. Co-expression of FLS2-YFPn and YFPc-StRem1.3 produced a heterogeneous distribution of the BiFC signal similar to that for Venus BiFC shown in Figure 1. (B) Transiently expressed in Arabidopsis mesophyll protoplasts, FLS2-StRem1.3 BiFC complexes exhibited heterogeneous discrete patches, different from either plasma membrane-localized FLS2-YFP or YFP-StRem1.3, or cytoplasm-localized YFP. Red fluorescence represents chloroplast autofluorescence. BF, bright field. Scale bars in (A,B) represent 10 μm. [file Image_2.JPEG]

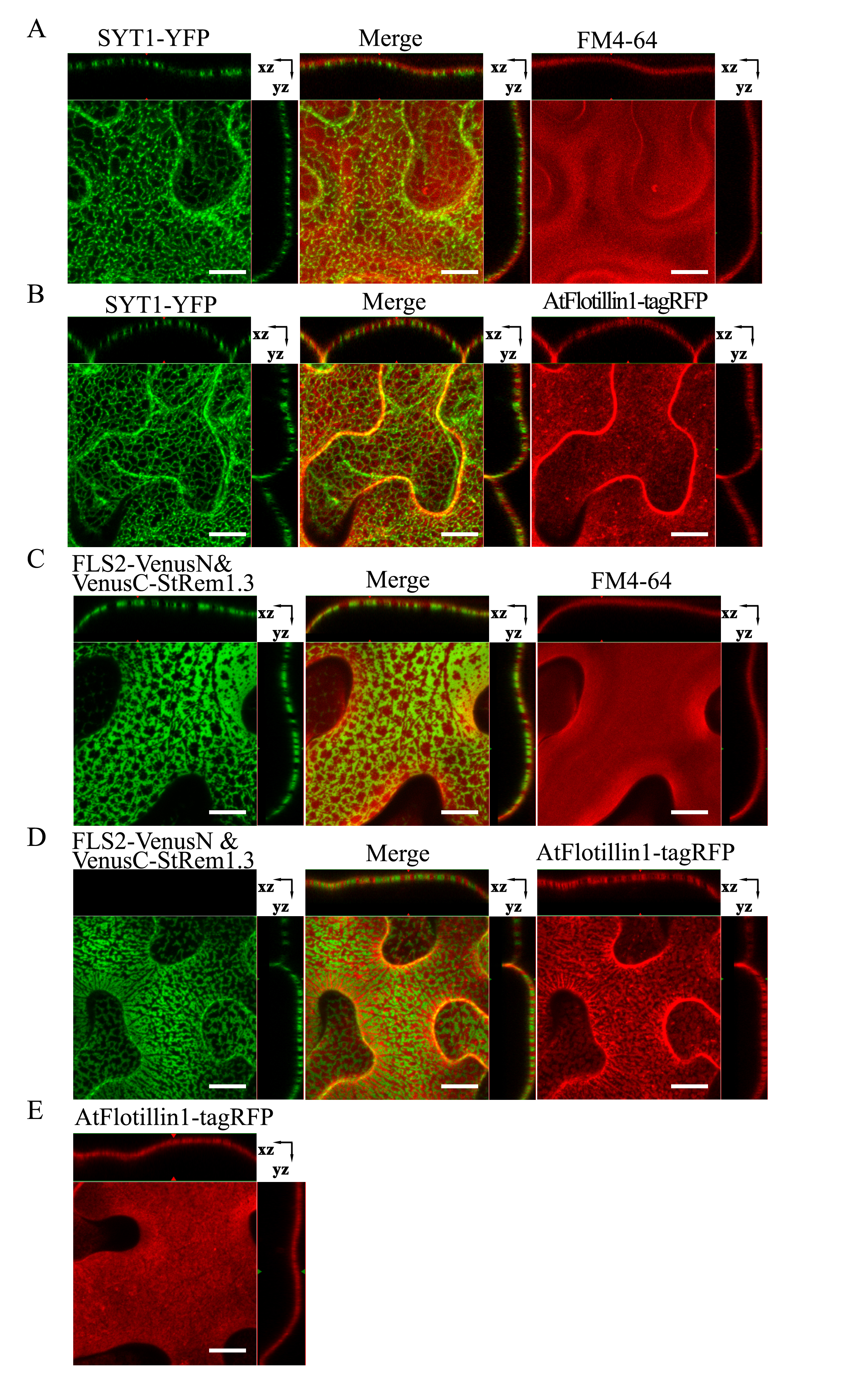

Supplement: Supplementary Figure S3 — Orthogonal imaging of SYT1 and FLS2-StRem1.3 BiFC complexes in N. benthamiana leaf cortical cells, relative to AtFlotillin1 and FM4-64. (A) The Arabidopsis tethering protein SYT1 tightly associates with the plasma membrane visualized by FM4-64. (B) Regions of the plasma membrane associated with SYT1 show reduced presence of membrane protein AtFlotillin1 fused to tagRFP. (C) FLS2-StRem1.3 BiFC complexes tightly associate with the plasma membrane, visualized by FM4-64. (D) Regions of the plasma membrane associated with FLS2-StRem1.3 BiFC complexes show reduced presence of AtFlotillin1-tagRFP. (E) Subcellular localization of AtFlotillin-1-tagRFP when expressed alone. Scale bars represent 10 μm. [file Image_3.JPEG]

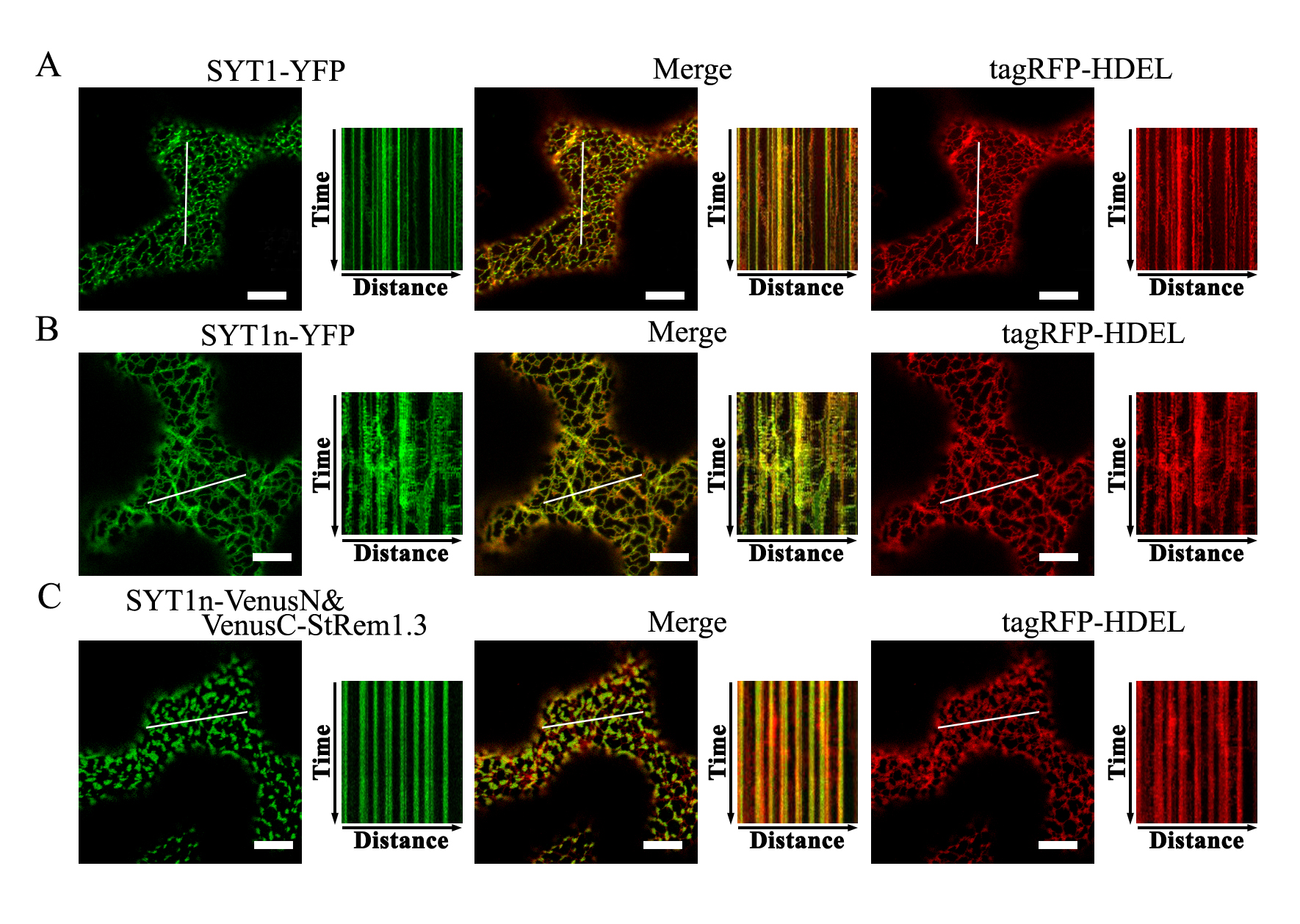

Supplement: Supplementary Figure S4 — Distribution and kymograph analysis of SYT1, SYT1n, and SYT1-StRem1.3 BiFC complexes co-expressed with ER marker SP-tagRFP-HDEL in N. benthamiana leaf cortical cells. (A) SYT1-YFP co-expressed with SP-tagRFP-HDEL. Arrowheads highlight bright puncta resembling ER-PM contact sites that stabilize the dynamic ER. (B) SYT1n-YFP co-expressed with SP-tagRFP-HDEL, showing coordinated dynamic mobility. (C) FLS2-StRem1.3 BiFC complexes co-expressed with SP-tagRFP-HDEL, showing stabilization of dynamic mobility. Kymographs produced as in Figure 2. Scale bars represent 10 μm. [file Image_4.JPEG]

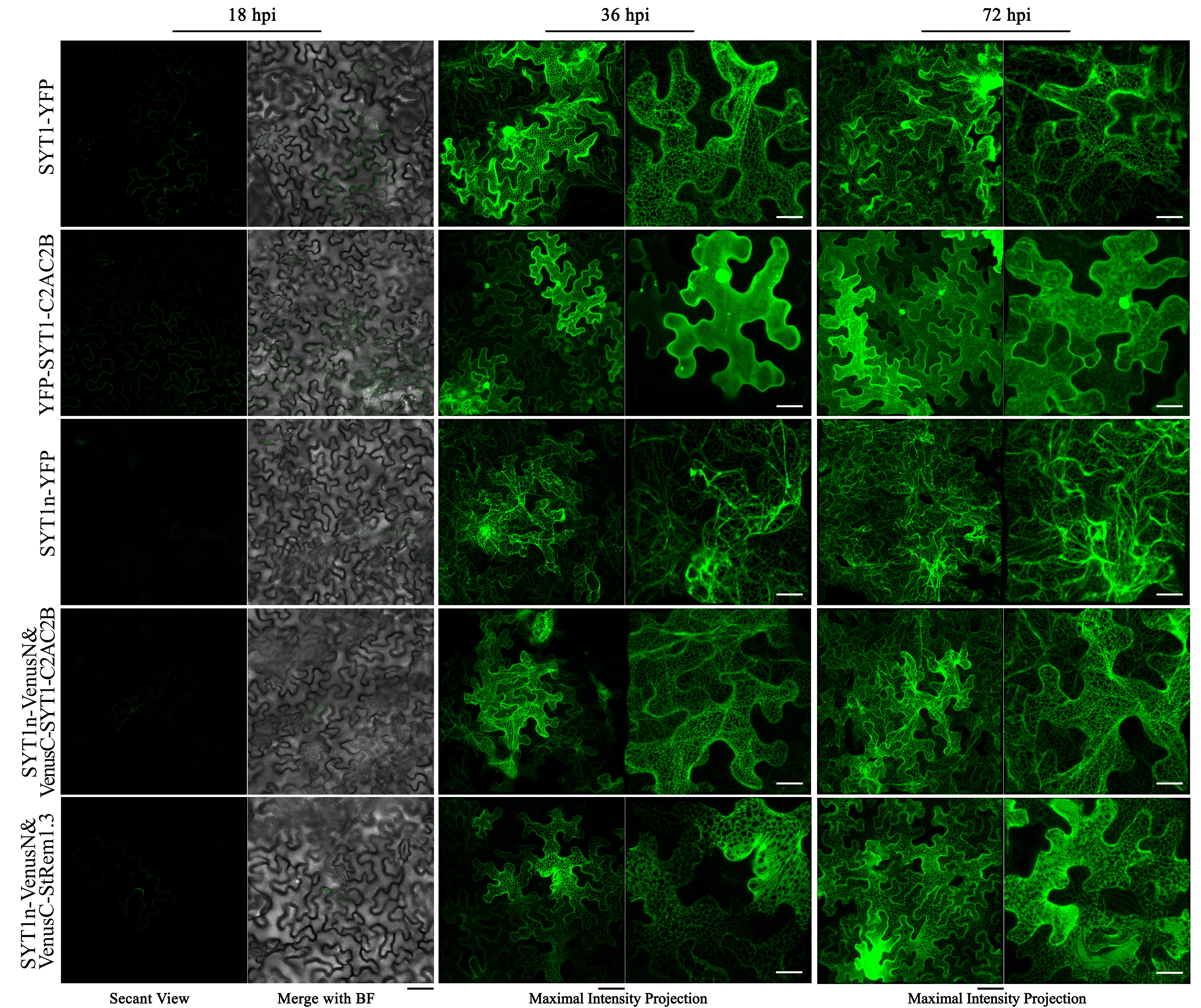

Supplement: Supplementary Figure S5 — Effect of expression time on the distribution of fluorescently tagged full length SYT1, C-terminal C2AC2B domain of SYT1, N-terminal transmembrane domain SYT1n, and BiFC complexes SYT1n&SYT1-C2AC2B, and SYT1n&StRem1.3 in N. benthamiana leaf cortical cells. Hpi = hours since Agrobacterium infiltration. Scale bars in black color represent 50 μm, and the white scale bars represent 20 μm. [file Image_5.JPEG]

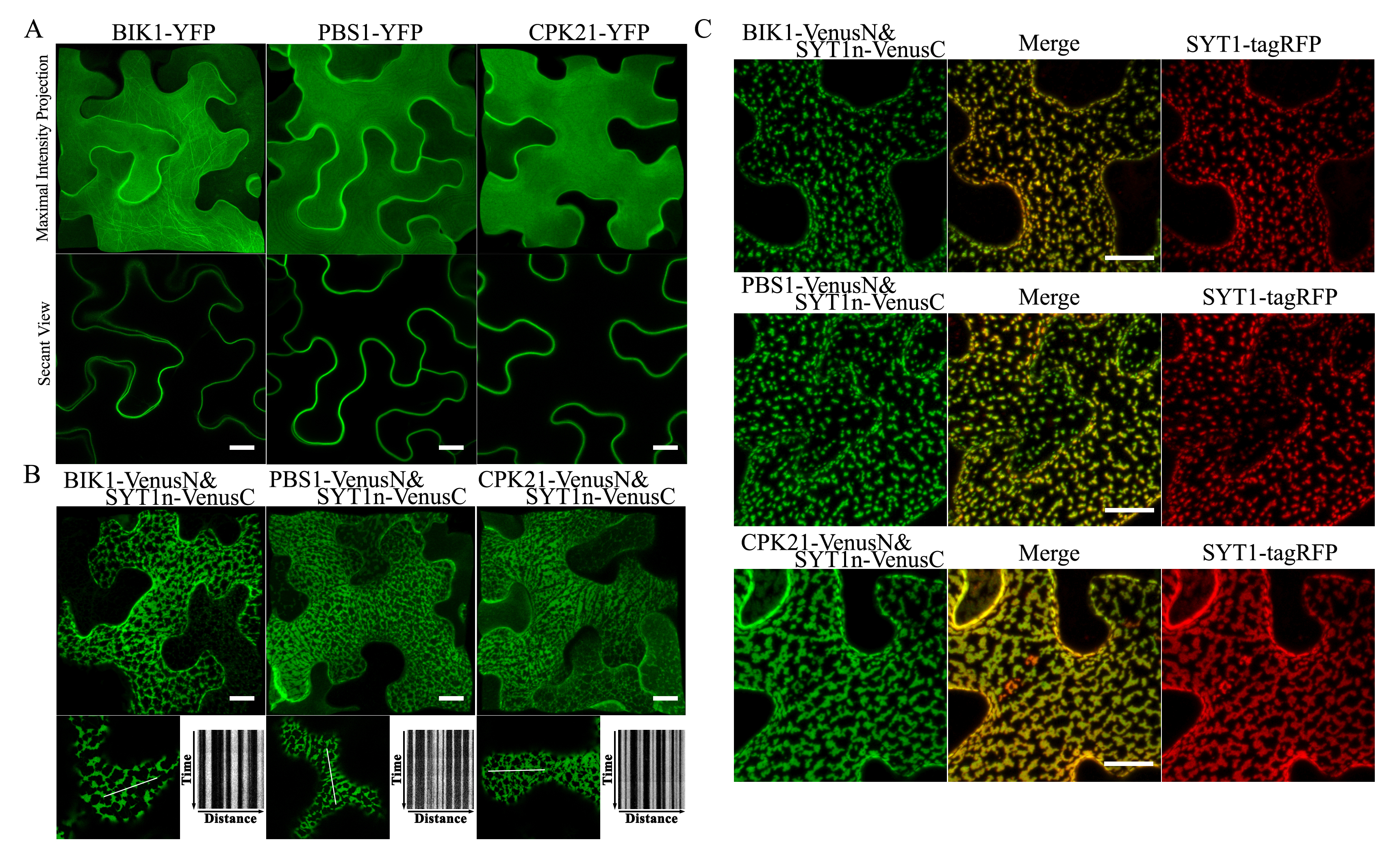

Supplement: Supplementary Figure S6 — Co-expression of peripheral membrane proteins and SYTn in BiFC complexes in N. benthamiana leaf cortical cells results in ER-PM tethering. (A) Distribution of peripheral membrane proteins BIK1, PBS1, and CPK21 fused with YFP. (B) Distribution and kymograph analysis of BiFC complexes produced by SYT1n-VenusC co-expressed with BIK1-VenusN, PBS1-VenusN, or CPK21-VenusN. (C) Puncta observed in BiFC complexes produced by BIK1 & SYT1n, PBS1 & SYT1n, and CPK21 & SYT1n co-localized with wild type SYT1-tagRFP. Kymographs produced as in Figure 2. Scale bars represent 10 μm. [file Image_6.JPEG]

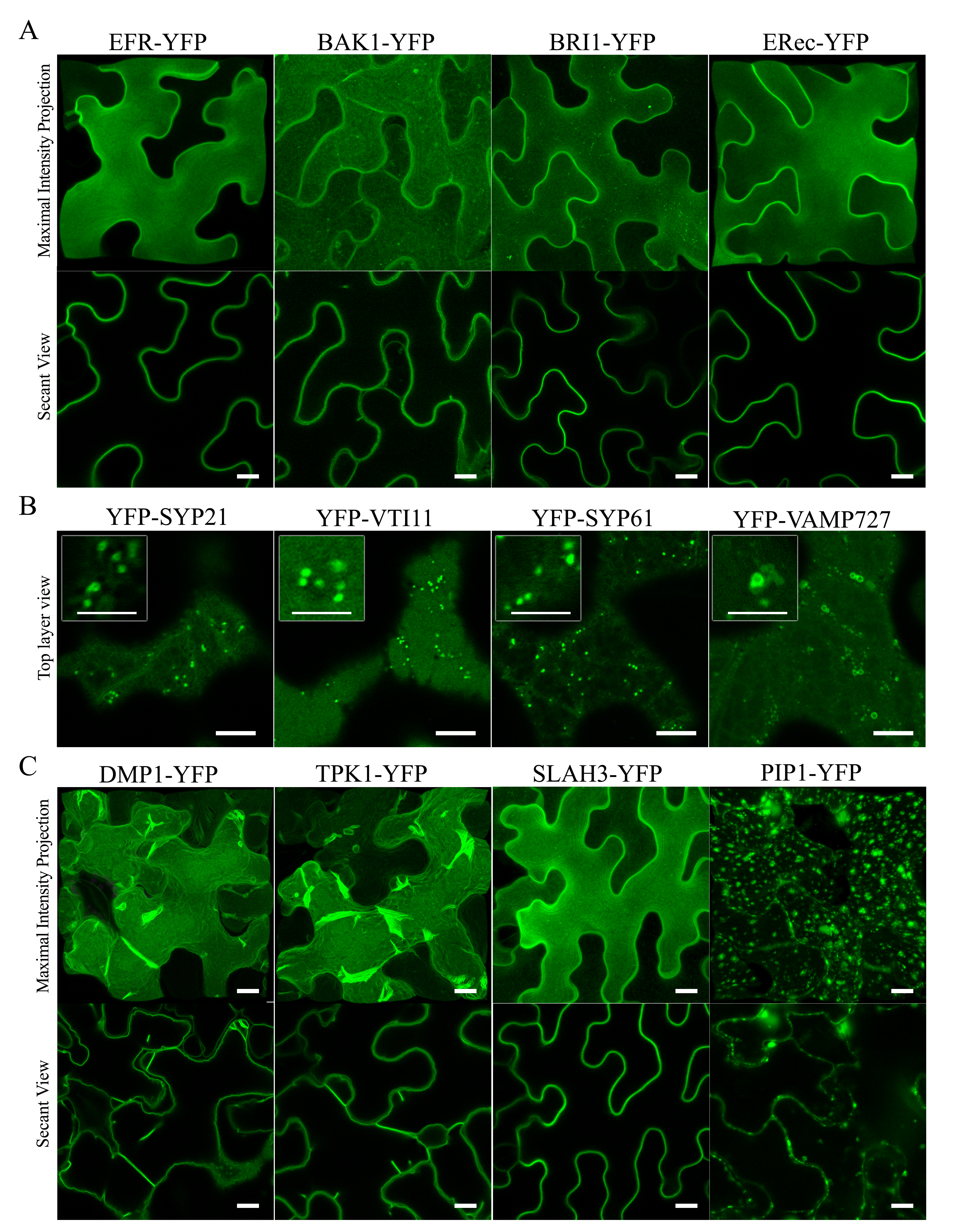

Supplement: Supplementary Figure S7 — Membrane distributions of integral membrane proteins fused to YFP and transiently expressed in N. benthamiana leaf cortical cells. (A) Subcellular localization of PM IMPs with a cleavable N-terminal signal peptide and single-pass TMD. (B) Subcellular localization of tail-anchored SNARE proteins. Qa-SNARE SYP21 and R-SNARE VAMP727 are localized to endosomal vesicles; Qb-SNARE VTI11 and Qc-SNARE SYP61 are localized to the Golgi. (C) Subcellular localization of IMPs with multi-pass TMDs. AtDMP1 and AtTPK1 are localized to the vacuolar membrane (tonoplast); SLAH3 is localized on the PM; PIP1 is localized on endosomal membranes. Scale bars in highlighted box represent 5 μm, and all others represent 10 μm. [file Image_7.JPEG]

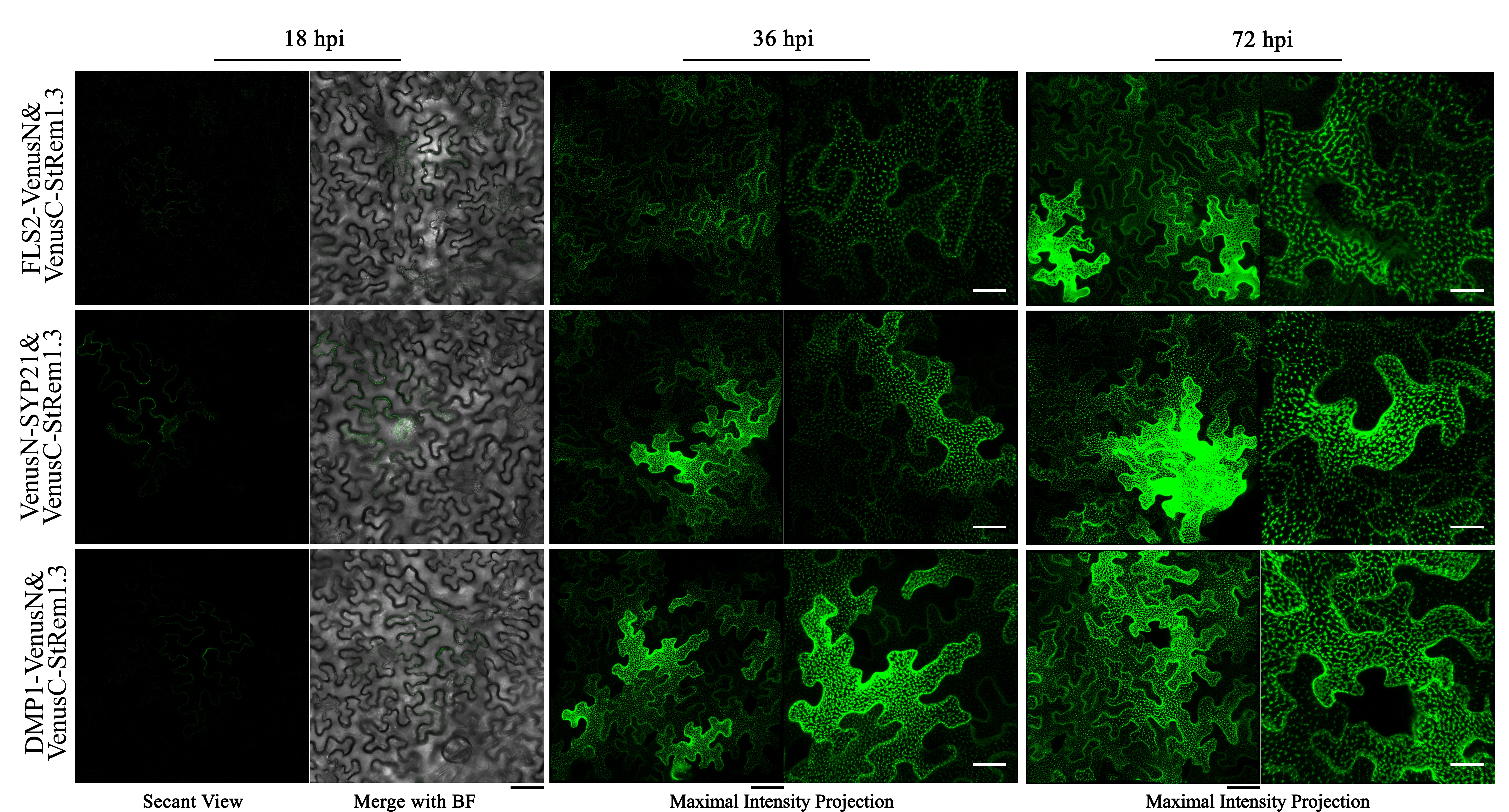

Supplement: Supplementary Figure S8 — Effect of expression time on the distribution of BiFC complexes FLS2-VenusN + VenusC-StRem1.3, VenusN-SYP21 + VenusC-StRem1.3, and DMP1-VenusN + VenusC-StRem1.3 in N. benthamiana leaf cortical cells. Scale bars in black color represent 50 μm, and the white scale bars represent 20 μm. Hpi = hours since Agrobacterium infiltration. [file Image_8.JPEG]

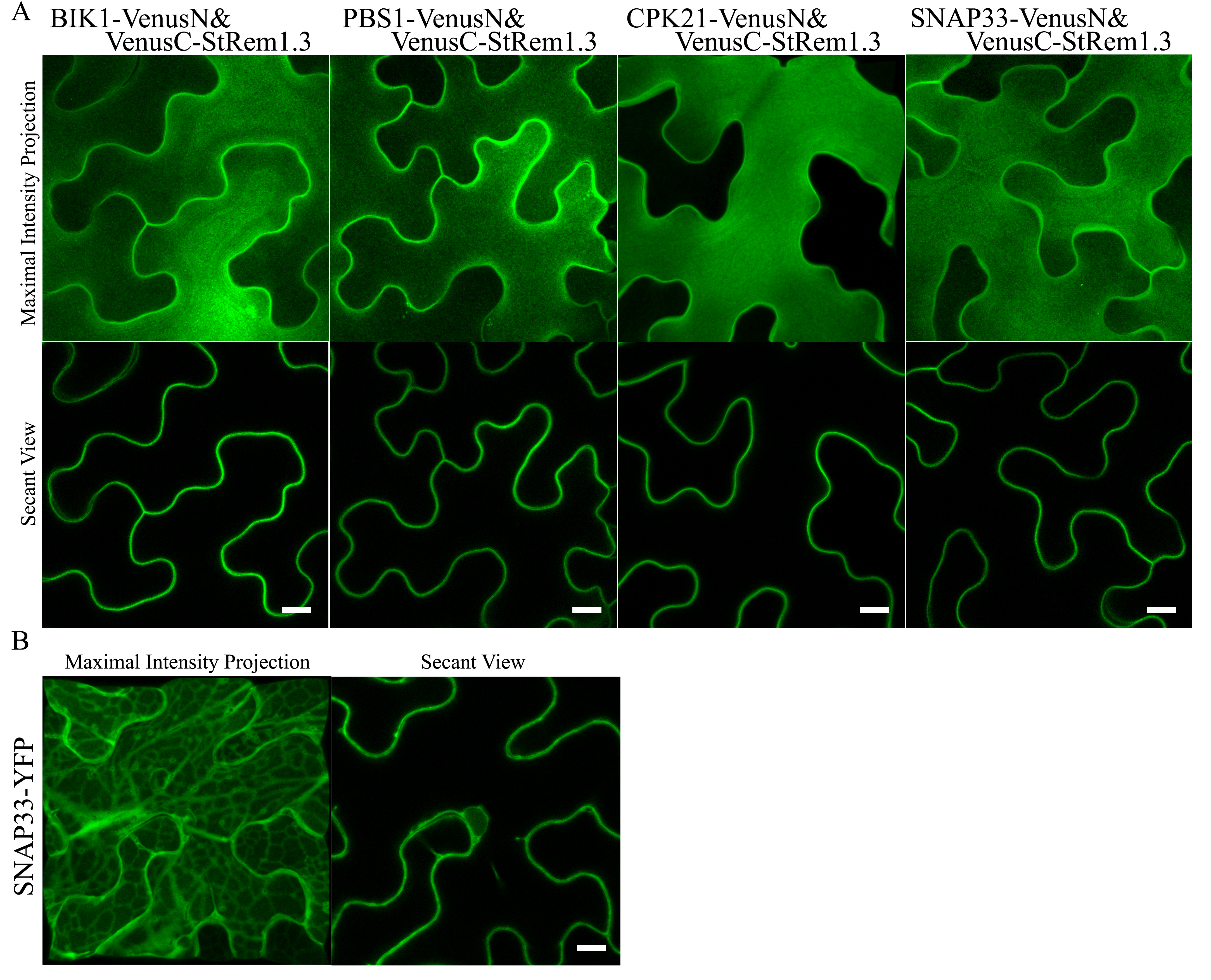

Supplement: Supplementary Figure S9 — Co-expression of peripheral membrane proteins and StRem1.3 in N. benthamiana leaf cortical cells does not result in ER-PM tethering by BiFC complexes. (A) Homogenously distributed fluorescent signal observed in BiFC complexes produced by BIK1 & StRem1.3, PBS1 & StRem1.3, CPK21 & StRem1.3, and SNAP33 & StRem1.3. (B) The subcellular localization of Qbc-SNARE protein SNAP33 that lacks a TMD. Scale bars represent 10 μm. [file Image_9.JPEG]

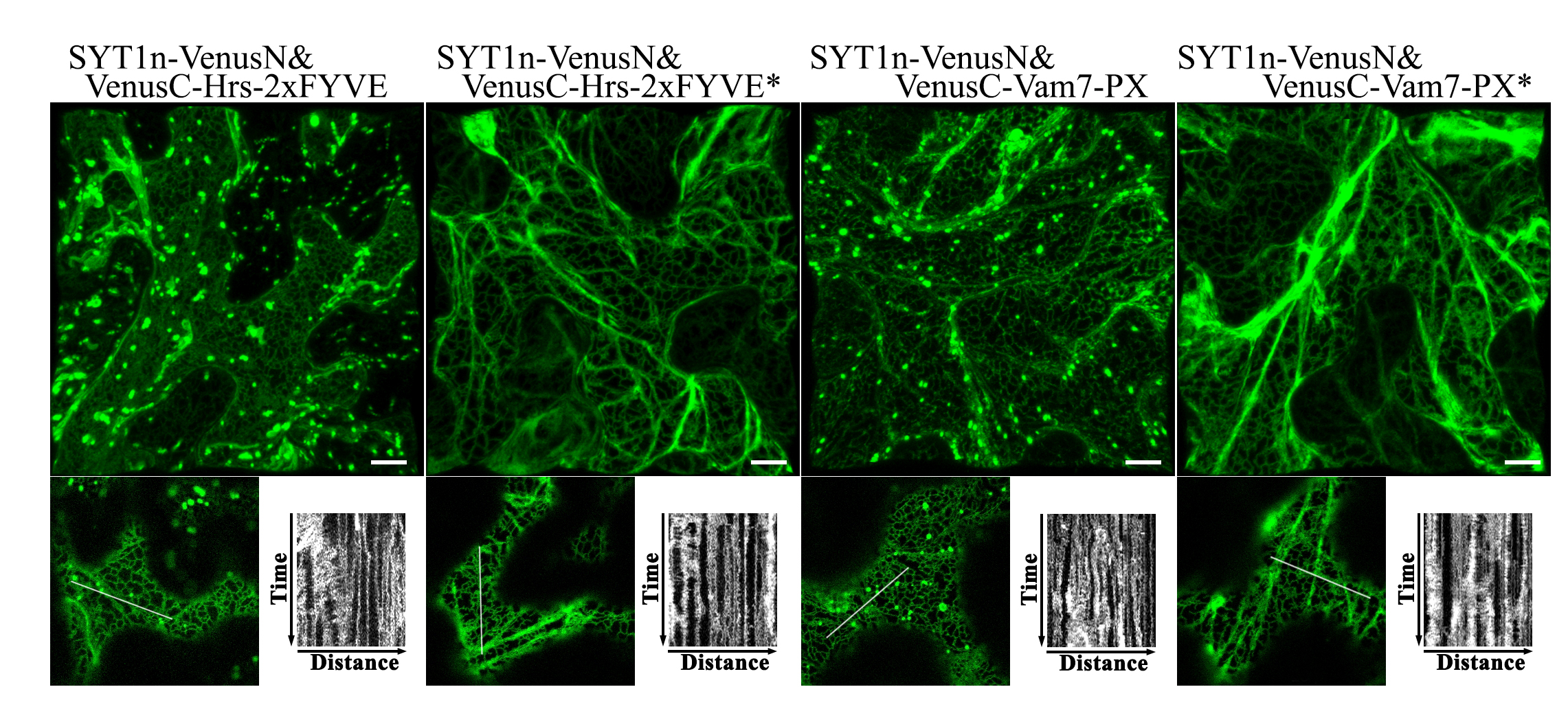

Supplement: Supplementary Figure S10 — Co-expression of PtdIns(3)P binding proteins with the SYT1 N-terminal domain in BiFC complexes in N. benthamiana leaf cortical cells does not result in ER-PM tethering. Hrs-2xFYVE, and Vam7-PX proteins are PtdIns(3)P binding proteins while Hrs-2xFYVE* and Vam7-PX* are PtdIns(3)P-non-binding mutants. Kymographs produced as in Figure 2. Scale bars represent 10 μm. [file Image_10.JPEG]
